# Nutrient availability controls the impact of mammalian herbivores on soil carbon and nitrogen pools in grasslands

**DOI:** 10.1111/gcb.15023

**Published:** 2020-02-24

**Authors:** Judith Sitters, E. R. Jasper Wubs, Elisabeth S. Bakker, Thomas W. Crowther, Peter B. Adler, Sumanta Bagchi, Jonathan D. Bakker, Lori Biederman, Elizabeth T. Borer, Elsa E. Cleland, Nico Eisenhauer, Jennifer Firn, Laureano Gherardi, Nicole Hagenah, Yann Hautier, Sarah E. Hobbie, Johannes M. H. Knops, Andrew S. MacDougall, Rebecca L. McCulley, Joslin L. Moore, Brent Mortensen, Pablo L. Peri, Suzanne M. Prober, Charlotte Riggs, Anita C. Risch, Martin Schütz, Eric W. Seabloom, Julia Siebert, Carly J. Stevens, G. F. (Ciska) Veen

**Affiliations:** ^1^ Department of Aquatic Ecology Netherlands Institute of Ecology (NIOO‐KNAW) Wageningen The Netherlands; ^2^ Department of Terrestrial Ecology Netherlands Institute of Ecology (NIOO‐KNAW) Wageningen The Netherlands; ^3^ Ecology and Biodiversity Department Biology Vrije Universiteit Brussel Brussels Belgium; ^4^ Sustainable Agroecosystems Group Institute of Agricultural Sciences Department of Environmental Systems Science ETH Zurich Zurich Switzerland; ^5^ Institute of Integrative Biology Department of Environmental Systems Science ETH Zurich Zurich Switzerland; ^6^ Department of Wildland Resources and the Ecology Center Utah State University Logan UT USA; ^7^ Centre for Ecological Sciences Indian Institute of Science Bangalore India; ^8^ School of Environmental and Forest Sciences University of Washington Seattle WA USA; ^9^ Department of Ecology, Evolution, and Organismal Biology Iowa State University Ames IA USA; ^10^ Department of Eology, Evolution, and Behavior University of Minnesota St. Paul MN USA; ^11^ Ecology, Behavior & Evolution Section University of California, San Diego La Jolla CA USA; ^12^ German Centre for Integrative Biodiversity Research (iDiv) Halle‐Jena‐Leipzig Leipzig Germany; ^13^ Institute of Biology Leipzig University Leipzig Germany; ^14^ Queensland University of Technology (QUT) Brisbane Qld Australia; ^15^ School of Life Sciences and Global Drylands Center Arizona State University Tempe AZ USA; ^16^ Mammal Research Institute Department of Zoology and Entomology University of Pretoria Pretoria South Africa; ^17^ Ecology and Biodiversity Group Department of Biology Utrecht University Utrecht The Netherlands; ^18^ Department of Health & Environmental Science Xi’an Jiaotong Liverpool University Suzhou China; ^19^ Department of Integrative Biology University of Guelph Guelph ON Canada; ^20^ Department of Plant & Soil Sciences University of Kentucky Lexington KY USA; ^21^ School of Biological Sciences Monash University Clayton Vic. Australia; ^22^ Department of Biology Benedictine College Atchison KS USA; ^23^ Instituto Nacional de Tecnología Agropecuaria (INTA) Rio Gallegos Argentina; ^24^ Universidad Nacional de la Patagonia Austral (UNPA)‐CONICET Rio Gallegos Argentina; ^25^ CSIRO Land and Water Wembley WA Australia; ^26^ Department of Soil, Water, and Climate University of Minnesota St. Paul MN USA; ^27^ Swiss Federal Institute for Forest, Snow and Landscape Research Birmensdorf Switzerland; ^28^ Lancaster Environment Centre Lancaster University Lancaster UK

**Keywords:** carbon sequestration, exclosure, fertilization, global change, grazing, herbivory, nutrient dynamics, nutrient enrichment, Nutrient Network (NutNet), soil microorganisms

## Abstract

Grasslands are subject to considerable alteration due to human activities globally, including widespread changes in populations and composition of large mammalian herbivores and elevated supply of nutrients. Grassland soils remain important reservoirs of carbon (C) and nitrogen (N). Herbivores may affect both C and N pools and these changes likely interact with increases in soil nutrient availability. Given the scale of grassland soil fluxes, such changes can have striking consequences for atmospheric C concentrations and the climate. Here, we use the Nutrient Network experiment to examine the responses of soil C and N pools to mammalian herbivore exclusion across 22 grasslands, under ambient and elevated nutrient availabilities (fertilized with NPK + micronutrients). We show that the impact of herbivore exclusion on soil C and N pools depends on fertilization. Under ambient nutrient conditions, we observed no effect of herbivore exclusion, but under elevated nutrient supply, pools are smaller upon herbivore exclusion. The highest mean soil C and N pools were found in grazed and fertilized plots. The decrease in soil C and N upon herbivore exclusion in combination with fertilization correlated with a decrease in aboveground plant biomass and microbial activity, indicating a reduced storage of organic matter and microbial residues as soil C and N. The response of soil C and N pools to herbivore exclusion was contingent on temperature – herbivores likely cause losses of C and N in colder sites and increases in warmer sites. Additionally, grasslands that contain mammalian herbivores have the potential to sequester more N under increased temperature variability and nutrient enrichment than ungrazed grasslands. Our study highlights the importance of conserving mammalian herbivore populations in grasslands worldwide. We need to incorporate local‐scale herbivory, and its interaction with nutrient enrichment and climate, within global‐scale models to better predict land–atmosphere interactions under future climate change.

## Graphical abstract

In this study, we use the Nutrient Network experiment to examine the responses of soil C and N pools to mammalian herbivore exclusion across 22 grasslands, under unfertilized and fertilized conditions. We show that the impact of herbivore exclusion on soil C and N pools depends on fertilization; the highest soil C and N pools were found in grazed (+H) and fertilized plots (+F). Our study highlights the importance of conserving mammalian herbivore populations in grasslands worldwide and incorporating local‐scale herbivory within global‐scale models to better predict land–atmosphere interactions under future climate change.

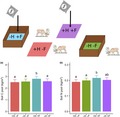

## 1 INTRODUCTION

Grasslands cover 30% of the terrestrial earth surface (White, Murray, & Rohweder, 2000) and their soils are important reservoirs of carbon (C) and nitrogen (N; Jobbagy & Jackson, 2000). Grasslands are subject to considerable ongoing alterations due to human activities (IPCC, 2014), including changes in populations and composition of grazing mammalian herbivores (Dirzo et al., 2014; Hempson, Archibald, & Bond, 2017; Ripple et al., 2015; Svenning et al., 2016). As nearly all natural grasslands co‐evolved with some degree of grazing (Axelrod, 1985; Janis, Damuth, & Theodor, 2002; Souttie, Reynold, & Batello, 2005) and mammalian herbivores are major drivers of grassland functioning (Blair, Nippert, & Briggs, 2014), changes in their populations or composition are expected to have important consequences for C and N fluxes and pools (McSherry & Ritchie, 2013; Pineiro, Paruelo, Oesterheld, & Jobbagy, 2010; Zhou et al., 2017). Given the scale of grassland soil fluxes, such changes can have striking consequences for atmospheric C concentrations and the climate; losses of soil C could exacerbate climate change whereas increased soil C sequestration may mitigate it (Crowther et al., 2016; Lu et al., 2013). As ecosystem C sequestration is constrained by nutrients (Crowther et al., 2019), and in particular N, changes in soil N are tightly linked to C feedbacks between land and atmosphere (Hungate, Dukes, Shaw, Luo, & Field, 2003).

**Figure 1:**
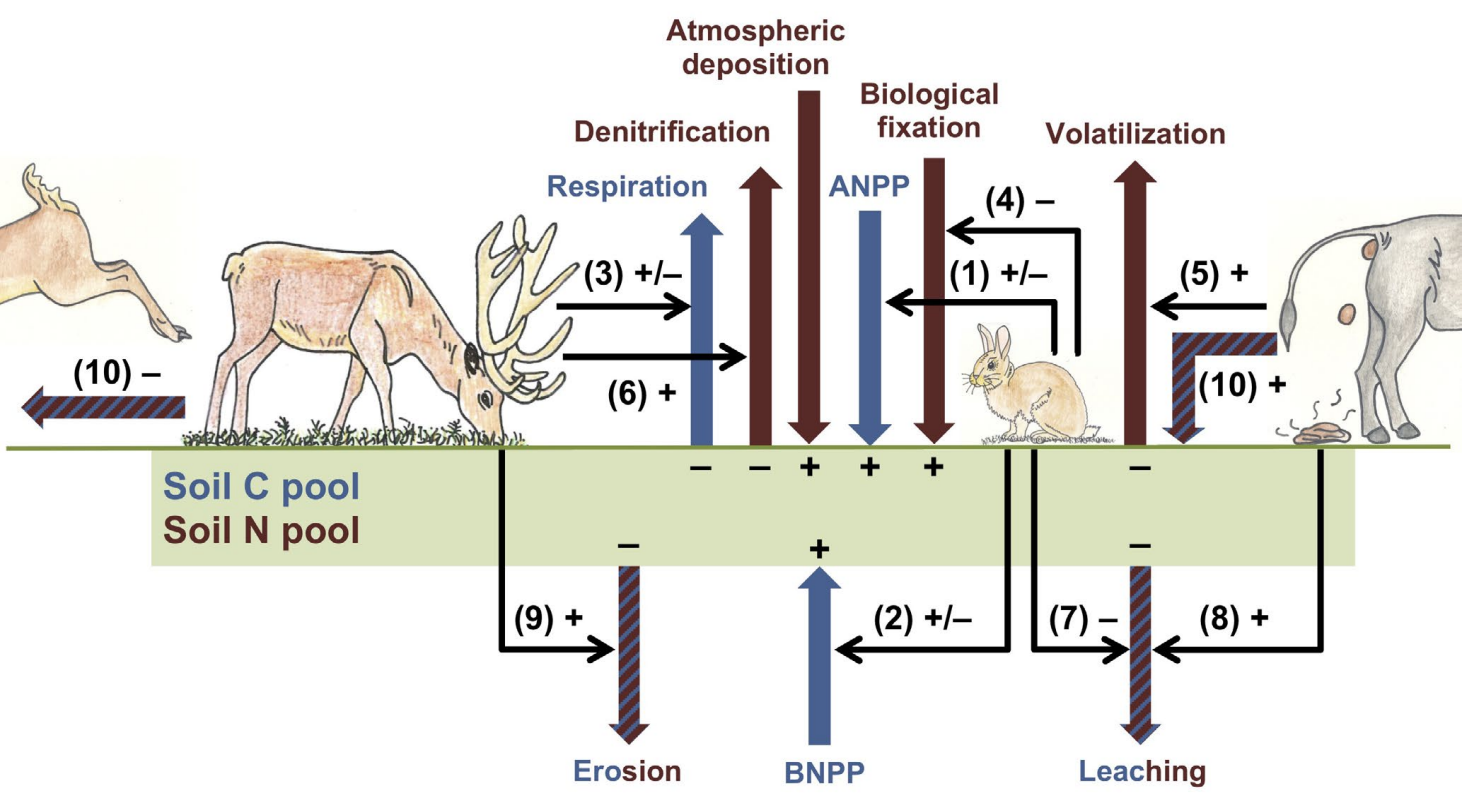
Conceptual framework showing how mammalian herbivores can influence soil C and N pools by their impact on C and N inputs to and outputs from the soil. The blue arrows are the main C fluxes and the brown arrows the main N fluxes, while the arrows shaded both blue and brown indicate both C and N fluxes. The thinner black arrows indicate the impact aboveground mammalian herbivores can have on these fluxes. Herbivores can modify C inputs to the soil by changing aboveground and belowground net primary production (ANPP and BNPP; arrows 1 and 2; Frank et al., 2002; Milchunas & Lauenroth, 1993; Pineiro et al., 2010; Ziter & MacDougall, 2013), thereby changing soil influx of litter and root exudates. C fluxes from the soil can be modified by herbivores via impacts on soil respiration rates and decomposition of organic matter (arrow 3), by changing the quantity and/or quality of organic inputs (dung, urine, plant litter), or through changes in soil conditions, such as temperature and moisture (Bardgett & Wardle, 2003; Pastor et al., 1993; Pineiro et al., 2010), and soil microbial communities and activity (Bardgett & Wardle, 2010). N input fluxes can be modified as herbivores generally reduce the biomass of N_2_‐fixing legumes (arrow 4; Ritchie & Tilman, 1995; Ritchie, Tilman, & Knops, 1998). They also may increase N losses by stimulating volatilization (arrow 5) via urine and dung deposition (Frank & Evans, 1997; Pineiro, Paruelo, Jobbagy, Jackson, & Oesterheld, 2009), denitrification (arrow 6) and surface runoff as a result of trampling‐induced soil compaction (Schrama et al., 2013), leaching (arrow 7) of mineral nutrients from urine and dung patches or soil erosion (arrow 9; Neff, Reynolds, Belnap, & Lamothe, 2005; Pei, Fu, & Wan, 2008; Steffens, Kolbl, Totsche, & Kogel‐Knabner, 2008; Steinauer & Collins, 2001). In contrast, C and N may be retained under herbivory (arrow 7) through greater plant root allocation (Derner, Boutton, & Briske, 2006; Pineiro et al., 2009; Reeder, Schuman, Morgan, & Lecain, 2004) and higher soil microbial activity (Lange et al., 2015). Herbivores can locally remove or add C and N (arrow 10), by feeding on plant biomass in one area, while depositing dung and/or urine in another (Giese et al., 2013; Singer & Schoenecker, 2003; Van Uytvanck, Milotic, & Hoffmann, 2010)

Herbivores influence the fluxes of C and N into and out of the soil locally, thereby determining soil C and N pools (Figure 1). Herbivores can alter C and N inputs to the soil by changing the quantity and quality of organic inputs (e.g. plant litter, herbivore dung), by decreasing biological fixation through the consumption of legumes, or through changes in soil conditions, such as temperature and moisture (Bardgett & Wardle, 2003; Pastor, Dewey, Naiman, McInnes, & Cohen, 1993; Pineiro et al., 2010), which in turn impact soil microbial communities and activity (Bardgett & Wardle, 2010). As herbivore‐induced changes in soil C and N fluxes occur simultaneously (Figure 1), considerable uncertainty exists regarding the net effect of herbivores on soil C and N pools, and which local C and N fluxes are most important in driving this herbivore effect. The direction and magnitude of the impact of herbivores on soil C and N have been shown to be contingent on environmental conditions, such as climate (e.g. temperature, rainfall), soil properties (e.g. fertility, texture) and site productivity (e.g. plant biomass; McSherry & Ritchie, 2013; Milchunas & Lauenroth, 1993; Pineiro et al., 2010; Schrama et al., 2013; Tanentzap & Coomes, 2012; Zhou et al., 2017). So far, however, studies on the effect of herbivores on soil C and N pools have not accounted for the effect of human‐induced increases in grassland soil nutrient availability, that may arise due to atmospheric N deposition or the use of artificial fertilizers (Asner et al., 2001; Galloway et al., 2004). This makes it difficult to incorporate the role of herbivores in global models predicting land–atmosphere interactions under future climate change.

Under increased nutrient inputs, the impact of herbivores on plant production (De Mazancourt, Loreau, & Abbadie, 1998; Ziter & MacDougall, 2013) and thereby C inputs into the soil may become more positive. Also, the capacity of grasses to regrow after herbivory may increase (Hawkes & Sullivan, 2001), allowing sustained C inputs belowground (Bardgett & Wardle, 2003; Hamilton & Frank, 2001; Olff & Ritchie, 1998), but only if grazing pressure is not too high (Zhou et al., 2017). In addition, attraction of herbivores to nutrient‐rich sites (van der Graaf, Stahl, Veen, Havinga, & Drent, 2007; van der Waal et al., 2011) will increase local biomass removal and waste inputs. Finally, increased nutrient availability may alleviate stoichiometric constraints for microorganisms (van Groenigen et al., 2017) resulting in greater microbial activity. Recent studies indicate that greater microbial acitivity might not only increase litter decomposition rates and C respiration, but alternatively increase C transfer into slow‐cycling forms of C (i.e. microbial necromass; Lange et al., 2015; Sokol & Bradford, 2019), thereby further increasing the potential for C sequestration under grazing. Hence, under future more eutrophied conditions it is likely that herbivores will promote soil C and N storage in grasslands.

Here, we quantify the responses of soil C and N pools to the exclusion of mammalian herbivores in 22 grasslands distributed across the globe, under both ambient and elevated nutrient supply. These sites are part of the Nutrient Network (NutNet) distributed experiment, which was established to examine the combined effects of nutrient addition and herbivore exclusion on ecosystem processes in grasslands worldwide (Borer, Grace, Harpole, MacDougall, & Seabloom, 2017). This collaborative experimental network uses a consistent and standardized methodology and experimental design. We use the experiment to: (a) test the responses of grassland soil C and N pools to herbivore exclusion and fertilization, (b) relate these responses to changes in plant biomass and soil microbial properties, and (c) examine if these responses are driven by environmental variables including climate, soil properties and site productivity.

## 2 METHODS

### 2.1 Site selection

For this study, we used sites from the Nutrient Network (http://www.nutnet.org; Borer, Harpole, et al., 2014), where each site consisted of three blocks of four treatment plots of 5 m × 5 m. Each block contained the following four treatment plots: (a) with herbivores and without fertilization (the unfenced control plot), (b) without herbivores and without fertilization (the fenced plot), (c) with herbivores and with fertilization (the fertilized plot), (d) without herbivores and with fertilization (the fenced and fertilized plot). At each block, two plots received no nutrients, while two plots received nitrogen (N), phosphorus (P) and potassium (K) plus micronutrients (μ) to alleviate all forms of nutrient limitation (Fay et al., 2015). The nutrient treatment involved annual application of 10 g m^−2^ year^−1^ N, P and K as time‐released urea [(NH_2_)_2_CO], triple‐super phosphate [Ca(H_2_PO_4_)_2_] and potassium sulphate [K_2_SO_4_] respectively. Once, at the start of the experiment, 100 g/m^2^ of μ mix of Fe (15%), S (14%), Mg (1.5%), Mn (2.5%), Cu (1%), Zn (1%), B (0.2%) and Mo (0.05%) was applied to all fertilized plots. At each block, one unfertilized and one fertilized plot were unfenced and subject to variable grazing by the contemporary suite of mammalian herbivores present per site (Table S2). The other unfertilized and fertilized plot were fenced (2.30 m high) to exclude aboveground mammalian herbivores (>c. 50 g). More details on the experimental set‐up and nutrient sources are available in Borer, Harpole, et al. (2014).

For this study, 22 NutNet sites were included (Table S1) as they each met two conditions: (a) soil property data had been collected (i.e. C and N concentrations, bulk density) in each of the four treatments for a minimum of 2 years of treatment applications (25 sites); and (b) mammalian herbivores were present in the sites and excluded by the fences (three sites were excluded by this criterion because herbivores were small, rare or absent). Hence, sites that only had herbivores with a body weight <c. 50 g (e.g. voles, mice, rats, squirrels, gophers) were not included. The mammalian herbivores in the selected sites ranged from domestic ungulates such as sheep to wild ungulates such as deer and wild macropods like kangaroos (for an overview of all herbivore species see Table S2). The majority of the herbivores in our sites were grazers or mixed‐feeders, no strict browsers were present, and therefore we describe herbivory in terms of ‘grazing’. Our study sites represent a wide range of herbaceous ecosystems including prairie, montane grassland, shrub steppe, alpine grassland and savanna. The sites also encompassed a wide range of environmental gradients including mean annual temperature (MAT; 0.1–18.2°C), mean annual precipitation (MAP; 246–1,877 mm) and total soil N concentration (0.06%–1.2%).

### 2.2 Soil C and N pools

After 2–4 years (3.5 years on average) of experimental nutrient addition and herbivore exclusion (Table S1), two soil cores (2.5 cm diameter at 10 cm depth) were collected from each plot after plant litter and vegetation were removed. The soil cores were sieved (2 mm) and homogenized per plot, air‐dried and analysed for total C and N content (Costech ESC 4010 Elemental Analyzer). At three sites where pH > 7.5, soil samples were pretreated with 0.1 M hydrochloric acid to remove carbonates (cdpt.us, hart.us, shps.us). In each plot, an additional intact soil core was collected, for which the volume of the core and fresh and dry weight of the soil were determined to estimate soil bulk density (for more details, see Data S1). To calculate soil C and N pools (kg/m^2^) in the top 10 cm, we multiplied values of soil bulk density with C and N concentrations for each plot. Grasslands store their greatest proportion of soil C and N near the soil surface (Crowther et al., 2016; Jobbagy & Jackson, 2001), but that does not preclude effects at depths that our sampling approach cannot account for.

We present the effect of the exclusion of mammalian herbivores (>50 g) as the log response ratio (RR) = ln(fenced/unfenced). We calculated separate RRs for the unfertilized and the fertilized (NPKμ) plots within each block per site. If RR = 0 herbivore exclusion had no effect on soil C or N, while RR < 0 indicates that herbivore exclusion decreased soil C or N and RR > 0 indicates that herbivore exclusion increased soil C or N.

### 2.3 Local controls of the impact of herbivore exclusion on soil C and N

We used data on the impact of herbivore exclusion on aboveground and belowground plant biomass and soil microbial properties collected at the plot‐level, to examine if changes in these local controls were related to the impact of herbivore exclusion on soil C and N (see Figure 1 for details on these potential mechanisms). Total aboveground biomass of all plants was clipped at peak biomass within two 1 m × 0.1 m strips. Aboveground biomass was sorted to live (current year's growth) and dead (previous years’ growth) biomass (or further into functional groups such as legumes), dried to a constant mass and weighed to the nearest 0.01 g. To accurately reflect the flux of biomass inputs to the soil from the start of the experiment up until the year the soil cores were collected, we calculated cumulative plant biomass for each plot by summing the annual harvested biomass over the years since the start of the experiment. This parameter therefore takes into account that longer running experiments might have greater C and N inputs from vegetation, possibly contributing to more pronounced changes in soil C and N.

Directly following aboveground biomass collection, five soil cores were collected from the clipped area, homogenized, and a subsample of the soil (c. 60 g) was used to estimate belowground plant biomass to a depth of 10 cm (Cleland et al., 2019). The subsample was suspended in water, and roots were captured with fine sieves and hand‐picked. Picked roots were dried at 40°C for 72 hr (to constant mass) and weighed to calculate dry root biomass per unit area.

In 2015, 12 of the 22 sites (Table S1) contributed samples for an additional research project focusing on soil microbial properties. At these sites three additional soil cores (5 cm diameter at 12 cm depth) were collected from each plot in 2015 to estimate microbial activity and biomass. Soils were sieved (2 mm) and homogenized per plot. An O_2_‐microcompensation system (Scheu, 1992) was used to measure the respiratory response of soil microbes in two separate steps using approximately 5.5 g of fresh soil. In a first step, basal respiration was determined as a measure of soil microbial activity (μl O_2_ hr^−1^ g^−1^ soil dry weight) without the addition of any substrate. In a second step, the maximal respiratory response to the addition of glucose solution (4 mg glucose/g soil dry weight dissolved in distilled water) allowed us to estimate soil microbial biomass (μg C_mic_/g soil dry weight; Anderson & Domsch, 1978).

Herbivore exclusion effects on all vegetation (live, dead and total aboveground biomass, legume biomass, root biomass) and microbial properties (activity, biomass) were estimated using log response ratios as RR = ln(fenced/unfenced), using both the unfertilized and fertilized plots separately in each block per site. Correlation analyses between all local controls were performed and when variables were strongly correlated (Pearson's *r* > .7), one of them was excluded to limit the impact of multicollinearity (see correlation Table S3). Threshold‐based preselection with a suggested threshold of .7 is an appropriate method to deal with collinearity between variables (Dormann et al., 2013). Hence, we excluded the impact of herbivore exclusion on total aboveground plant biomass (live + dead), as this variable was highly correlated with the impact of herbivore exclusion on live biomass. Thus, we retained four—or six in the subset of sites including microbial data—candidate local controls.

### 2.4 Environmental drivers of the impact of herbivore exclusion on soil C and N

We used data on climate and several vegetation and soil properties at the site‐level as candidate environmental drivers of the impact of herbivore exclusion on soil C and N. We selected six climate variables from the WorldClim database (version 1.4; Hijmans, Cameron, Parra, Jones, & Jarvis, 2005) that summarized the mean and seasonality of temperature and rainfall and their seasonal synchrony (Seabloom et al., 2013): (a) MAT (°C), (b) temperature seasonality (*SD* of temperature among months; TEMP_VAR), (c) mean annual range in temperature (°C; ANN_TEMP_RANGE), (d) mean temperature of wettest quarter (°C; TEMP_WET_Q), (e) MAP (mm), and (f) precipitation seasonality (coefficient of variation in precipitation among months; MAP_VAR). These global climate data were interpolated at high spatial resolution from weather/meteorological stations with 10–30 years of data (Hijmans et al., 2005). To determine the annual atmospheric N deposition (kg N ha^−1^ year^−1^) for each site, we used modelled rates based on existing measurements using a global three‐dimensional chemistry‐transport model (TM3, The Oak Ridge National Laboratory Distributed Active Archive Center; http://daac.ornl.gov/). The model provides sufficient spatial resolution (50 × 50 km grid cells) to distinguish site‐level variation in annual N deposition among our sites (Borer, Seabloom, et al., 2014).

We included data on aboveground plant biomass, soil N and soil texture at each site before the NutNet experimental treatments were established (pretreatment data at year 0), to get an accurate measure of the in situ productivity, soil fertility and texture before herbivore presence and site fertility were manipulated. We calculated mean values for the unfertilized and fertilized treatment per block at each site, by averaging the values of the unfenced and fenced treatment plots per fertilization treatment. Pretreatment soil N concentrations were missing for the fertilized plots in two sites (mtca.au and sgs.us), so means were based on the unfertilized plots (assuming no large differences between plots in year 0). Soil texture (% sand, silt and clay) was measured in one plot per block using the hydrometer method (Ashworth, Keyes, Kirk, & Lessard, 2001). Based on correlation analyses between all environmental drivers we excluded mean annual range in temperature, as this variable was highly correlated with temperature seasonality (Table S4). We therefore retained 10 candidate environmental drivers.

### 2.5 Statistical analyses

We excluded one block of data from the site smith.us because of missing soil data on the fertilized (+NPKμ) treatment, which precluded calculation of the response ratio. Additionally, one block in sage.us was dropped because of an extremely low bulk density value in its unfenced control plot (0.04 g/cm^3^ compared to a mean of 0.66 g/cm^3^ for this treatment in the other two blocks) due to waterlogging (95.9% soil moisture) and was therefore considered to be an outlier. All statistical analyses were therefore performed on data from 252 plots in 63 blocks (three blocks per site except for bldr.us, smith.us and sage.us) in 22 sites (Table S1).

To examine the impact of herbivore exclusion and fertilization on soil C and N pools, we used linear mixed models (LMMs) with block nested within site as random effect. In addition, we performed one sample *t* tests on our RRs to examine the impact of the exclusion of herbivores on soil C and N pools and C:N ratio under unfertilized and fertilized conditions. If the 95% confidence interval values of the RRs did not overlap with zero, there was a significant decrease or increase with herbivore exclusion. We also tested if treatment duration affected the RRs using an LMM, with number of treatment years as fixed predictor and site ID as a random effect. We found no significant impact of treatment duration on the responses of soil C and N pools to herbivore exclusion (LMM, *F*_1,20_ = 0.65, *p* = .431 for C and *F*_1,20_ = 1.30, *p* = .268 for N), allowing us to pool the data across treatment years, even though the sites differed in the number of years that the treatments were applied.

We used multi‐model inference (Burnham & Anderson, 2002; Richards, Whittingham, & Stephens, 2011) to examine (a) which local controls over soil C and N were responsible for changes in soil C and N pools due to herbivore exclusion, and (b) which across‐site environmental drivers affected the impact of herbivore exclusion on soil C and N pools. For this, we modelled the effects of our predictor variables (either the local controls or the environmental variables) on the C and N response ratios with a full LMM with site ID as a random effect. The models also included fertilization as a fixed factor to observe any significant interactions between fertilization and local controls/environmental drivers. Multi‐model inference uses model averaging based on Akaike's information criterion (AIC) to arrive at consistent parameter estimates of the most important explanatory variables in the full LMM, by averaging a set of top models which share similarly high levels of parsimony. We defined the top models as those that fell within 4 AIC units of the model with the lowest AIC value (Richards et al., 2011). We standardized our regression predictors through centering and dividing by 2 *SD* (Gelman, 2008), which resulted in variance inflation factors <5 of all predictors, indicating low collinearity among them (Dormann et al., 2013).

We assessed the response of soil C and N to herbivore exclusion with and without the impact of herbivore exclusion on legume biomass (local control) and with and without soil texture (environmental driver) as we missed data on these variables at several sites. These variables had no significant effect on the response of soil C and N to herbivore exclusion, and to maintain the largest spatial extent possible, we only present models without these variables. Additionally, we ran a separate model on the subset of sites including microbial data (12 of 22 sites; Table S1), and results of this model are also presented.

## 3 RESULTS

**Figure 2:**
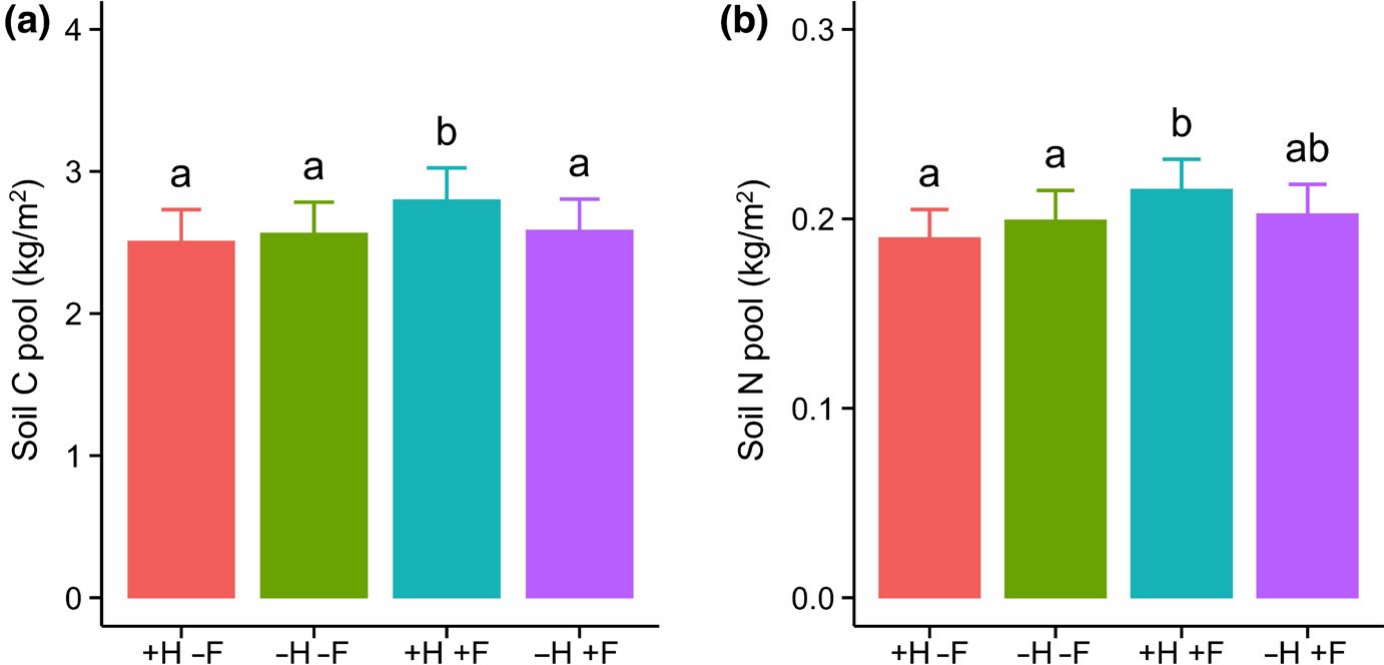
Effect of herbivore exclusion (+H: herbivores present; −H: herbivores excluded) and fertilization (+F: fertilized with NPKµ; −F: unfertilized) on soil C (a) and N pools (b). Shown are sample means ± *SE*. Different letters indicate significant differences among the treatment means

**Figure 3:**
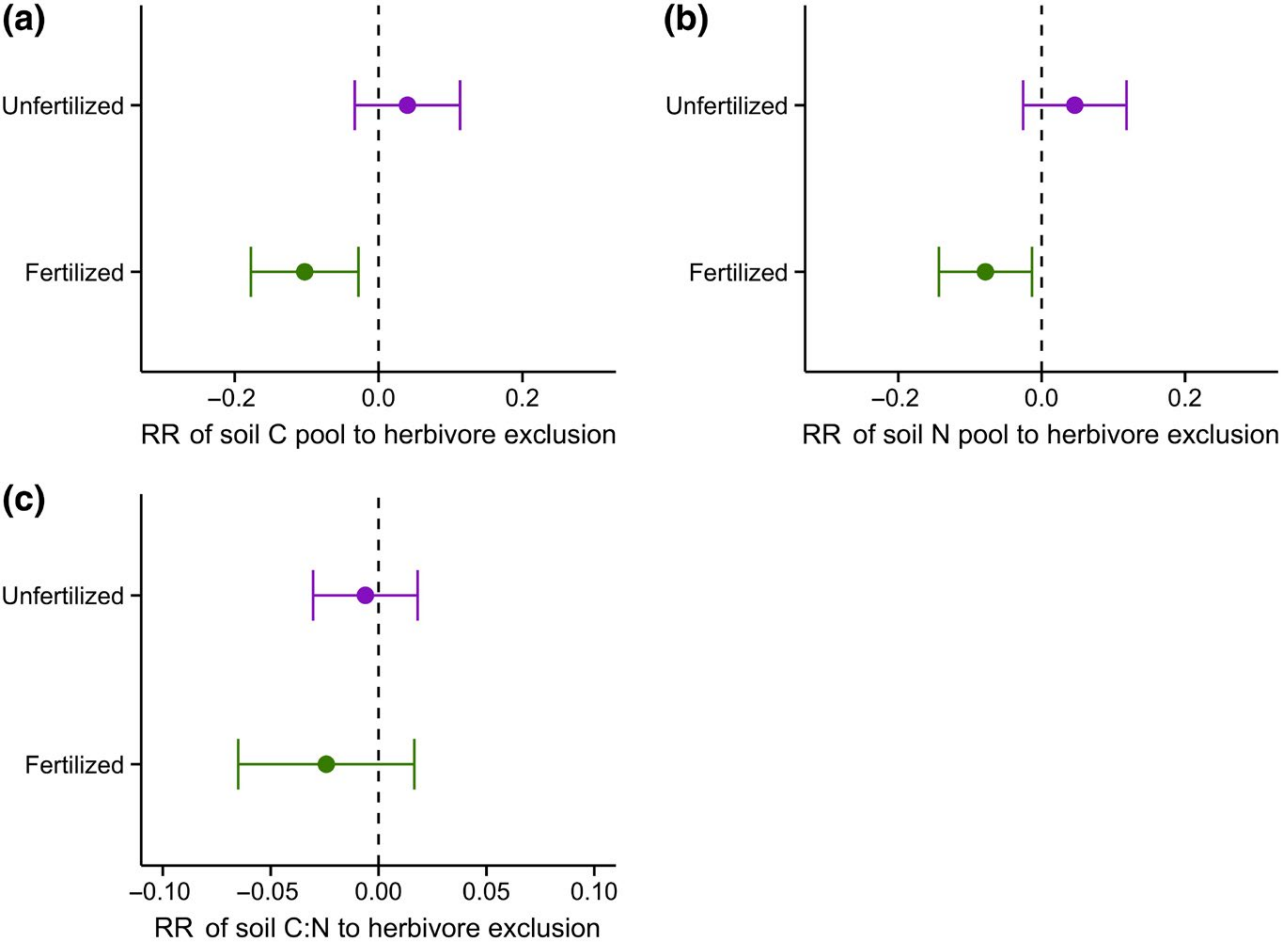
Log response ratios of soil C pool (a), N pool (b) and C:N ratio (c) to herbivore exclusion calculated as RR = ln(fenced/unfenced) for unfertilized (purple) and fertilized (NPKμ) plots (green). If response ratio (RR) = 0 herbivore exclusion had no effect on the variable, while RR < 0 herbivore exclusion decreased the variable and RR > 0 herbivore exclusion increased the variable. Graphs show the mean RRs across all 22 sites (*n* = 63 per fertilization treatment), where points represent the mean RR and error bars represent the range of 95% confidence intervals. The vertical dashed line was drawn at RR = 0 and responses are considered significant if error bars do not overlap with zero

Fertilization led to significant increases in soil C and N pools, but only when herbivores were not excluded (LMM, *F*_3,186_ = 6.46, *p* < .001 for C and *F*_3,186_ = 7.12, *p* < .001 for N; Figure 2). In fertilized plots, this led to herbivore exclosures having C pools that were on average 61.0 g m^−2^ year^−1^ (2.2%/year) smaller and N pools that were on average 3.7 g m^−2^ year^−1^ (1.7%/year) smaller (*p* = .005 for C and *p* = .01 for N; Figure 3a,b) than in grazed plots. Without fertilization, herbivore exclusion had no effect on soil C and N pools (*p* = .14 for C and *p* = .11 for N). Herbivore exclusion had no impact on soil C:N ratio under unfertilized or fertilized conditions (Figure 3c). The responses of soil C and N concentrations to herbivore exclusion and fertilization showed similar patterns as the C and N pools (*p* = .02 for C and *p* = .04 for N under fertilized conditions), while there was no effect on soil bulk density (Figure S1).

**Figure 4:**
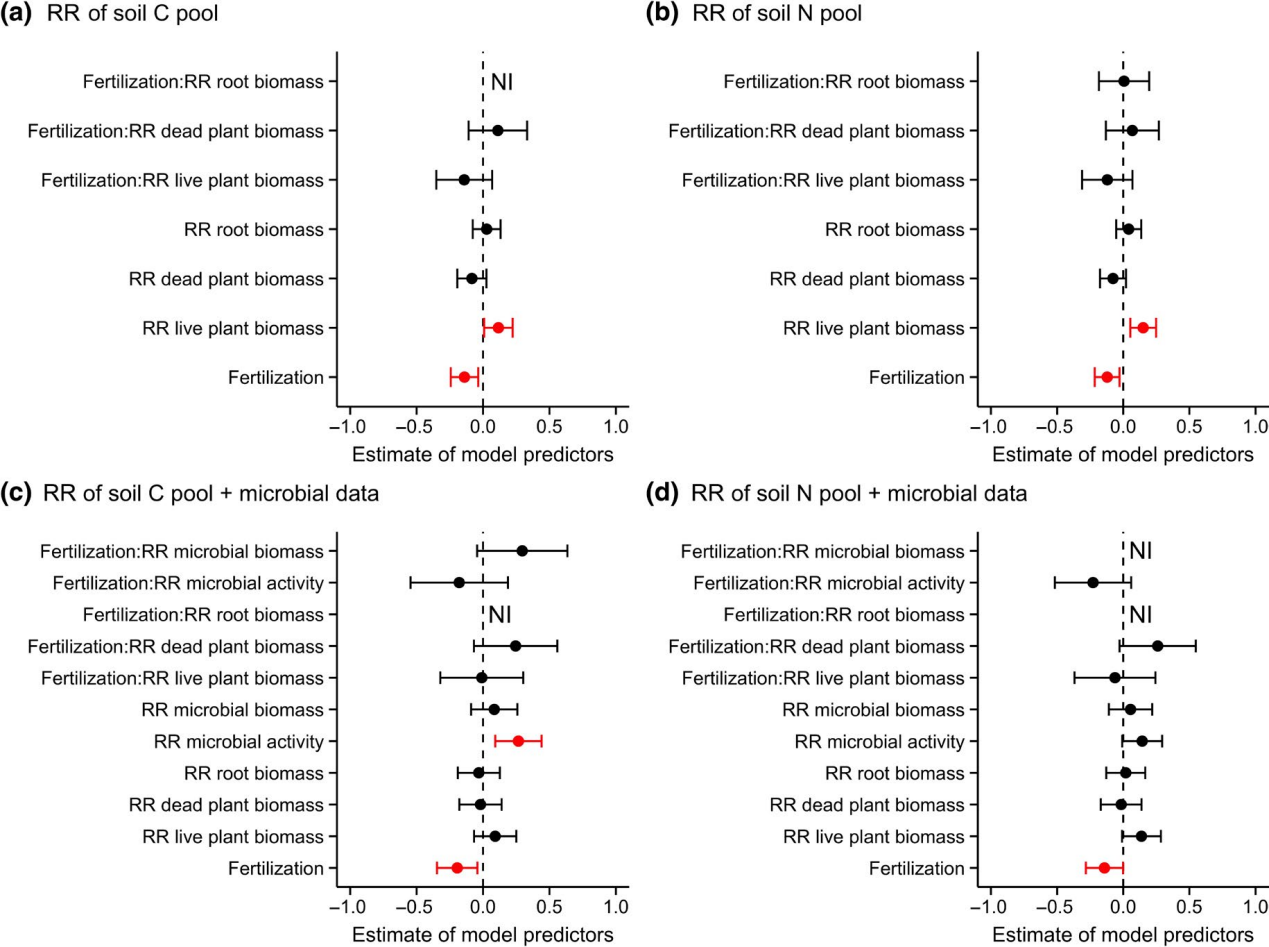
Plots showing the parameter estimates of the potential local controls explaining the response ratios (RR) of soil C (a, c) and N pools (b, d) to herbivore exclusion. The parameters are response ratios of plant biomass (live, dead and root) and microbial properties (biomass, activity) to herbivore exclusion. Parameter estimates were generated by multi‐model inference, which uses model averaging to arrive at consistent parameter estimates of the most important explanatory variables. Models included fertilization as a fixed factor (under fertilization the effect of herbivore exclusion is negative; also see Figure 3) and interactions are presented as ‘Fertilization:other parameter’. Models were run without (22 sites, *n* = 126; a, b) and with (12 sites, *n* = 67; c, d) microbial data. Points represent the mean value of the model predictor while error bars represent the range of 95% confidence intervals. Predictors are considered significant if error bars do not overlap with zero and are coloured red. NI indicates the variable was not included in the set of top models

The between‐site variation in the responses of soil C and N pools to herbivore exclusion was positively, albeit weakly, correlated with changes in aboveground live plant biomass (Figure 4a,b; Table S5; model‐averaged *R* ^2^ for all predictor variables = .09 for C and .12 for N); when herbivore exclusion increased plant biomass, it also increased soil C and N pools, and when herbivore exclusion decreased plant biomass, it also decreased soil C and N pools (Figure S2a,b). Fertilization did not have an impact on this relationship (i.e. no significant interaction). We also found a positive correlation with changes in microbial activity; when exclusion of herbivores increased microbial activity it also increased soil C pools and tended to increase soil N pools (it did significantly increase soil N concentrations; Figure 4b,c; Figure S2c; Table S5). This relationship was again not impacted by fertilization. Our results differed slightly for the analyses with and without the microbial data, possibly because only a subset of sites collected soil microbial data. We detected no overall effect of herbivore exclusion on aboveground live plant biomass or microbial activity (Figure S3). We did not find any detectable relationship between responses of soil C and N pools to herbivore exclusion and changes in dead plant biomass or root biomass (Figure 4; Table S5).

**Figure 5:**
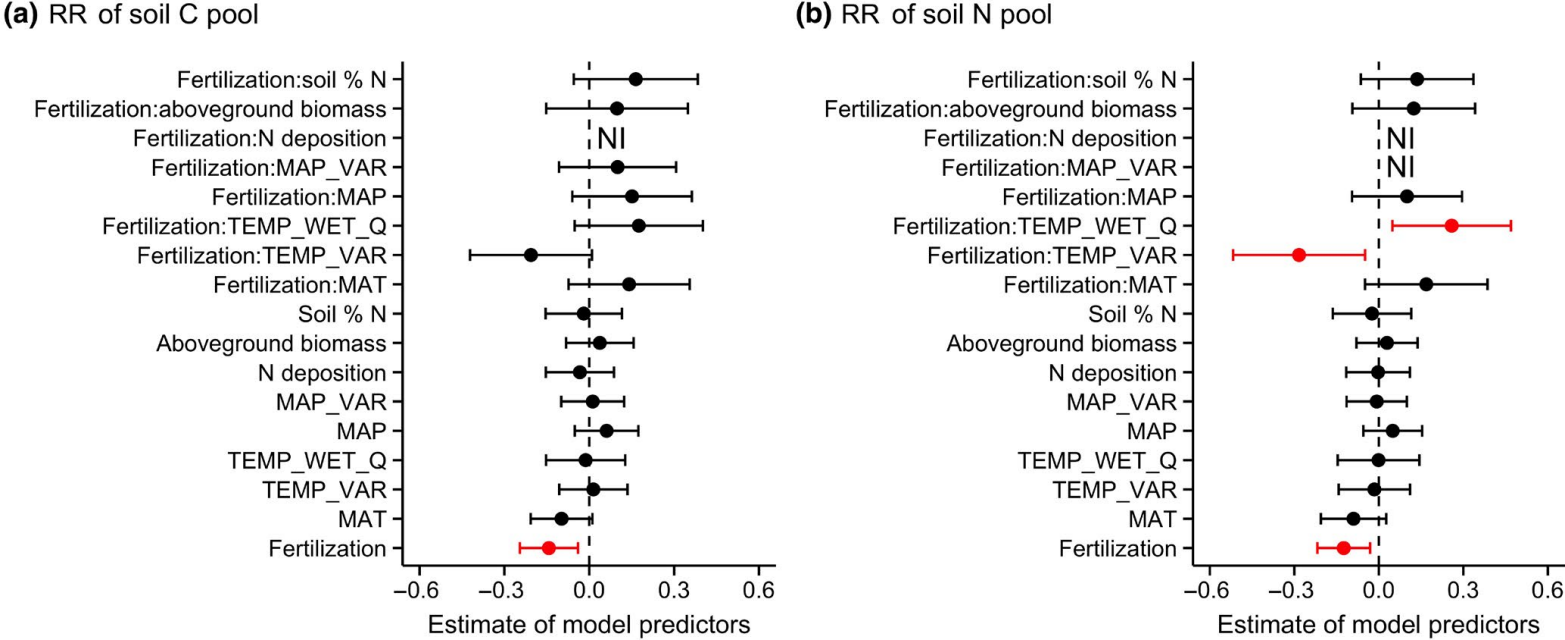
Plots showing the parameter estimates of the potential environmental drivers explaining the response ratios (RR) of soil C (a) and N pools (b) to herbivore exclusion. Parameter codes are: MAT, mean annual temperature; TEMP_VAR, temperature seasonality; TEMP_WET_Q, mean temperature of wettest quarter; MAP, mean annual precipitation; MAP_VAR, precipitation seasonality; aboveground biomass and soil % N are measures of in situ productivity and soil fertility. See Section 2 for more details. Parameter estimates were generated by multi‐model inference, which uses model averaging to arrive at consistent parameter estimates of the most important explanatory variables. Models included fertilization as a fixed factor and interactions are presented as ‘Fertilization:other parameter’. Points represent the mean value of the model predictor while error bars represent the range of 95% confidence intervals. Predictors are considered significant if error bars do not overlap with zero and are coloured red. NI indicates the variable was not included in the set of top models

Across sites, variation in temperature, precipitation, N deposition, aboveground plant biomass or soil fertility had minimal predictive power in explaining the variation in the impact of herbivore exclusion on soil C (model‐averaged *R* ^2^ = .09) or N pools (model‐averaged *R* ^2^ = .12; Figure 5; Table S6). However, the soil N response to herbivore exclusion was related to several climate variables in interaction with fertilization, indicating that these relationships were controlled by nutrient addition. Significant interactions were observed between fertilization and temperature seasonality (TEMP_VAR), and between fertilization and mean temperature of wettest quarter (TEMP_WET_Q; Figure 5b; Table S6). These interactions indicate that, in fertilized plots, the decrease of soil N pools upon herbivore exclusion increased with temperature variability among months (TEMP_VAR; Figure S4a), while it decreased with temperature in the wettest quarter (TEMP_WET_Q; Figure S4b). Additionally, the impact of herbivore exclusion on soil C and N concentrations became increasingly negative with increasing MAT, and the same pattern was observed for soil C and N pools, although it was not significant (Table S6).

## 4 DISCUSSION

Overall, nutrient availability explained most strongly the responses of soil C and N pools to the exclusion of mammalian herbivores: under ambient nutrient conditions, we observed no effect of herbivore exclusion, but under elevated nutrient supply (fertilization with NPKμ) pools were smaller upon herbivore exclusion (Figure 3). This means that fertilized plots that were grazed had the highest soil C and N pools (Figure 2). These results demonstrate that nutrient availability is a limiting factor in grazer‐induced C and N sequestration in grasslands across a broad range of environmental conditions. As soil C and N covaried in their response to herbivore exclusion, the soil C:N ratio was less variable (Figure 3c), suggesting that soil C:N ratio is well‐constrained in our grassland sites regardless of herbivory and nutrient availability. Indeed, the average ratio of 13.0 ± 0.2 was very similar to the average C:N ratio of 13.8 ± 0.4 across 75 grasslands on a larger scale (Cleveland & Liptzin, 2007), indicating stoichiometric balance despite variation in total soil C and N.

Previous clipping experiments combined with nutrient addition demonstrated an increase in grassland soil C storage, which was related to increased belowground production (Frank, Kuns, & Guido, 2002; Ziter & MacDougall, 2013; Figure 1). However, this mechanism was not associated with C storage in our grassland sites (Figure 4a), although this might be because our biomass measures did not capture root turnover (Frank et al., 2002). Alternatively, herbivores might be attracted to the fertilized plots due to increased aboveground plant biomass (Fay et al., 2015) and forage quality (La Pierre & Smith, 2015), resulting in increases in C and N inputs through dung and urine (van der Graaf et al., 2007; van der Waal et al., 2011; Figure 1). Carbon contained in herbivore dung can then be incorporated into the soil due to the activities of invertebrates, such as dung beetles or termites, thus allowing little C to volatize (Ritchie, 2014). Moreover, in fertilized and grazed plots, plants are likely to regrow better after herbivory (Hawkes & Sullivan, 2001), allowing for increased root exudation and C inputs belowground (Bardgett & Wardle, 2003; Hamilton & Frank, 2001; Figure 1). Grazing also may increase the photosynthetic rates of the regrowth and residual plant biomass, especially under high nutrient supply, leading to higher relative growth rates and increased productivity (Frank & McNaughton, 1993; McNaughton, 1985). Although we observed an increase in aboveground plant biomass due to fertilization, the extent of this increase did not differ between exclosures and grazed plots (Figure S3). It is possible that increased biomass removal by herbivores in the fertilized and grazed plots in combination with increased plant growth resulted in an overall net effect of zero. To confirm this, we would need to estimate annual net primary productivity and biomass consumption in our NutNet plots (e.g. using moveable exclosures; McNaughton, Milchunas, & Frank, 1996).

Our findings suggest that when herbivore exclusion increased aboveground plant biomass, this was related to an increase in soil C and N pools, while when herbivore exclusion decreased biomass, for example because of the disappearance of compensatory plant growth responses due to the absence of grazing, it might decrease plant inputs into the soil and thereby C and N pools (Figures 1 and 4a,b; Figure S2a,b; Frank, McNaughton, & Tracy, 1998; Milchunas & Lauenroth, 1993; Pineiro et al., 2010). In addition, we found that a decrease in microbial activity due to herbivore exclusion, potentially mediated via shifts in litter and dung inputs and root turnover (Ziter & MacDougall, 2013), was associated with a decreased soil C pool (Figure 4c; Figure S2c). Our results are in line with recent studies showing that decreased microbial activity results in reduced storage of microbial residues as soil C, suggesting that microbial activity could serve as a proxy for C transfer into slow‐cycling forms of C (Lange et al., 2015; Sokol & Bradford, 2019). Changes in legume biomass due to herbivore exclusion did not have an impact on the response of soil N, suggesting that the decrease of N input by legumes through selective feeding by herbivores might not be very common in grasslands (Ritchie & Tilman, 1995; Figure 1). Even though herbivore‐induced changes in aboveground plant biomass and microbial activity may affect changes in soil C and N pools, the amount of variation that these factors explained was relatively low (<25%; Table S5). This highlights the limitation of the local factors we were able to measure and suggests that other local (unmeasured) factors, e.g. volatilization and leaching (Figure 1), are likely important in explaining impacts of herbivore exclusion on soil C and N. Moreover, the microbial activity as measured here might not be the best index of C balance in the soil; to further unravel the role of microbes in driving the responses of soil C and N to herbivore exclusion additional measurements on microbial carbon use efficiency and microbial residues in the soil organic matter are recommended (Geyer, Kyker‐Snowman, Grandy, & Frey, 2016; Manzoni et al., 2018).

Fertilization not only controlled the responses of soil C and N to herbivore exclusion, but also modified the relationship of soil N response to interannual temperature variability (Figure 5b). Our results indicate that, with increased temperature variability, grasslands that contain herbivores have the potential to sequester more N under nutrient enrichment. Additionally, in warmer grasslands (higher MAT) the presence of herbivores could increase soil C and N sequestration (Table S6). In contrast, herbivore presence may stimulate losses of C and N in colder sites, which are already more likely to lose a considerable amount of C to the atmosphere as a result of global warming (Crowther et al., 2016). This relationship with temperature could be related to a shift from C_3_‐ to C_4_‐dominated grasslands, whereby grazing increases soil C in C_4_‐dominated grasslands likely due to an increase in belowground production of root C and/or mycorrhizae (McSherry & Ritchie, 2013). We however did not find an effect of grass type on the response of soil C to herbivore exclusion, which might be related to the disproportionate distribution of the grass types among our sites (Figure S5). Our study highlights the importance of conserving herbivore populations in grasslands, as the capacity of herbivores to enable soils to store more C and N is likely to become stronger under global change (i.e. under nutrient enrichment, increased temperature and interannual temperature variability). Additionally, the presence of herbivores might make grasslands better able to withstand climate change, as increases in soil C and N storage could result in increases in productivity and soil water‐holding capacity (Teague et al., 2011).

Studies have acknowledged that herbivore type and grazing intensity are important factors in determining the effects of herbivore exclusion on soil C and N pools (Bakker, Olff, Boekhoff, Gleichman, & Berendse, 2004; Pastor, Cohen, & Hobbs, 2006; Zhou et al., 2017). We did not have data on the density of the different herbivore species in our sites, however we could estimate grazing intensity (see Seabloom et al., 2013). We found little evidence that this grazing intensity estimate explained responses of soil C or N to herbivore exclusion (Figure S6). Future data collection on herbivores at each site using standardized methods will support further insight into the ecosystem effects of different types and densities of herbivores. However, we stress that despite the high among‐site variability in herbivore species (Table S2) and proxied grazing intensity (Figure S7), and hence variability in responses of soil C and N to herbivore exclusion across sites (Figure S8), we detected a significant overall decrease in soil C and N under fertilized conditions when herbivores were excluded (Figure 3). This points to a consistent, general effect that rises above the ‘noise’ from among‐site variation in herbivores. We captured these net changes in C and N dynamics over a relatively short time‐period (c. 3.5 years), and expect these changes to become greater with time (Fornara & Tilman, 2012). Additionally, our results are likely to be a conservative representation of soil C and N pools as we only sampled to a depth of 10 cm, while, for example, C accumulation can be substantial below these depths (Jobbagy & Jackson, 2000). At larger spatial scales the interactive effects of herbivores and nutrient enrichment remain uncertain. If grasslands were enriched with nutrients on a landscape‐scale, for instance through increased atmospheric deposition, the effect of herbivore attraction to local patches might likely disappear, but the overall carrying capacity of the ecosystem for herbivores could increase (i.e. increase in quantity and quality of forage; Parsons, Rowarth, Thornley, & Newton, 2011), potentially resulting in an increase of herbivore impacts that could facilitate soil C and N sequestration.

Here, we show that nutrient enrichment in concert with herbivore grazing is likely to increase total organic matter storage in grasslands, especially where herbivore exclusion is associated with a decrease in aboveground live biomass and/or decreased microbial activity. The broad‐scale experiment enabled us to examine the environmental contingencies of these soil C and N responses, suggesting that these effects might be greatest in warmer regions, and will increase with climate change. This work highlights the importance of incorporating local‐scale herbivory, and its interaction with nutrient enrichment and climate, within global‐scale models predicting land–atmosphere interactions under future climate change (Crowther et al., 2019; Schmitz et al., 2014; Thebault, Mariotte, Lortie, & MacDougall, 2014; Tylianakis, Didham, Bascompte, & Wardle, 2008).

## AUTHOR CONTRIBUTIONS

This specific study was designed by Judith Sitters, E.R.J.W., E.S.B., T.W.C. and G.F.V. The NutNet experiment was coordinated by E.T.B., S.E.H. and E.W.S. Statistical analyses were performed by Judith Sitters and E.R.J.W., with additional data and analytical contributions from E.E.C., N.E., C.R. and Julia Siebert. Data collection was performed by P.B.A., J.D.B., L.B., E.T.B., E.E.C., N.E., J.M.H.K., R.L.M., J.L.M., S.M.P., C.R., A.C.R., M.S., E.W.S. and Julia Siebert. Judith Sitters and E.R.J.W. wrote the first draft of the manuscript and all authors contributed substantially to revisions. See Table S7 for details.

## Data Availability

Data associated with this study are available from the Dryad Data Repository https://doi.org/10.5061/dryad.wstqjq2gw.
