# Supplementary material for: Nutrient availability controls the impact of mammalian herbivores on soil carbon and nitrogen pools in grasslands
Source: Glob Chang Biol. 2020 Feb 24;26(4):2060–71. doi: 10.1111/gcb.15023 (PMC7155038; doi:10.1111/gcb.15023)

**Supplementary Information for:**

**Nutrient availability controls the impact of mammalian herbivores on soil carbon and nitrogen pools in grasslands**

Judith Sitters^1,2,3^*^#^, E.R. Jasper Wubs^2,4#^, Elisabeth S. Bakker^1^, Thomas W. Crowther^2,5^, Peter B. Adler^6^, Sumanta Bagchi^7^, Jonathan D. Bakker^8^, Lori Biederman^9^, Elizabeth T. Borer^10^, Elsa E. Cleland^11^, Nico Eisenhauer^12,13^, Jennifer Firn^14^, Laureano Gherardi^15^, Nicole Hagenah^16^, Yann Hautier^17^, Sarah E. Hobbie^10^, Johannes M. H. Knops^18^, Andrew S. MacDougall^19^, Rebecca L. McCulley^20^, Joslin L. Moore^21^, Brent Mortensen^22^, Pablo L. Peri^23,24^, Suzanne M. Prober^25^, Charlotte Riggs^26^, Anita C. Risch^27^, Martin Schütz^27^, Eric W. Seabloom^10^, Julia Siebert^12,13^, Carly J. Stevens^28^, G.F. (Ciska) Veen^2^

^1^ Department of Aquatic Ecology, Netherlands Institute of Ecology (NIOO-KNAW), P.O. Box 50, 6700 AB, Wageningen, the Netherlands, ^2^ Department of Terrestrial Ecology, Netherlands Institute of Ecology (NIOO-KNAW), P.O. Box 50, 6700 AB, Wageningen, the Netherlands, ^3^ Ecology and Biodiversity, Department Biology, Vrije Universiteit Brussel, 1050 Brussels, Belgium, ^4^ Sustainable Agroecosystems Group, Institute of Agricultural Sciences, Department of Environmental Systems Science, ETH Zurich, Zurich, Switzerland, ^5^ Institute of Integrative Biology, Department of Environmental Systems Science, ETH Zurich, Zurich, Switzerland, ^6^ Department of Wildland Resources and the Ecology Center, Utah State University, Logan, UT 84103, USA, ^7^ Centre for Ecological Sciences, Indian Institute of Science, Bangalore 560012, India, ^8^ School of Environmental and Forest Sciences, University of Washington, Seattle, Washington 98195, USA, ^9^ Department of Ecology, Evolution, and Organismal Biology, Iowa State University, Ames, IA 50010, USA, ^10^ Department of Eology, Evolution, and Behavior, University of Minnesota, St. Paul, MN 55108, USA, ^11^ Ecology, Behavior & Evolution Section, University of California, La Jolla, San Diego, CA 92093, USA, ^12^ German Centre for Integrative Biodiversity Research (iDiv), Halle-Jena-Leipzig, 04103 Leipzig, Germany, ^13^ Leipzig University, Institute of Biology, 04103 Leipzig, Germany, ^14^ Queensland University of Technology (QUT), Gardens Point Campus, Brisbane Queensland, 40000, Australia, ^15^ School of Life Sciences and Global Drylands Center, Arizona State University, Tempe, AZ 85281, USA, ^16^ Mammal Research Institute, Department of Zoology and Entomology, University of Pretoria, Pretoria, South Africa, ^17^ Ecology and Biodiversity Group, Department of Biology, Utrecht University, Utrecht, CH 3584, The Netherlands, ^18^ Department of Health & Environmental Science, Xi’an Jiaotong Liverpool University, Suzhou, 215123, China, ^19^ Department of Integrative Biology, University of Guelph, Guelph, Ontario, Canada N1G 2W1, ^20^ Department of Plant & Soil Sciences, University of Kentucky, Lexington, KY 40546, USA, ^21^ School of Biologcal Sciences, Monash University, Clayton Campus, VIC 3800, Australia, ^22^ Department of Biology, Benedictine College, Atchison, KS 66002, USA, ^23^ Instituto Nacional de Tecnología Agropecuaria (INTA), 9400 Río Gallegos, Rio Gallegos, Argentina, ^24^ Universidad Nacional de la Patagonia Austral (UNPA)-CONICET, 9400 Río Gallegos, Rio Gallegos, Argentina, ^25^ CSIRO Land and Water, Private Bag 5, Wembley, Western Australia, 6913, Australia, ^26^ Department of Soil, Water, and Climate, University of Minnesota, St. Paul, MN 55108 USA, ^27^ Swiss Federal Institute for Forest, Snow and Landscape Research, Zuercherstrasse 111, 8903 Birmensdorf, Switzerland, ^28^ Lancaster Environment Centre, Lancaster University, Lancaster, LA1 4YQ, UK

^#^These authors contributed equally to this work

*Corresponding author: judith.sitters@vub.be, +32-2-6293496

**This PDF file includes:**

Supplementary Figs. S1 to S8

Supplementary Tables S1 to S8

Supplement S1

**
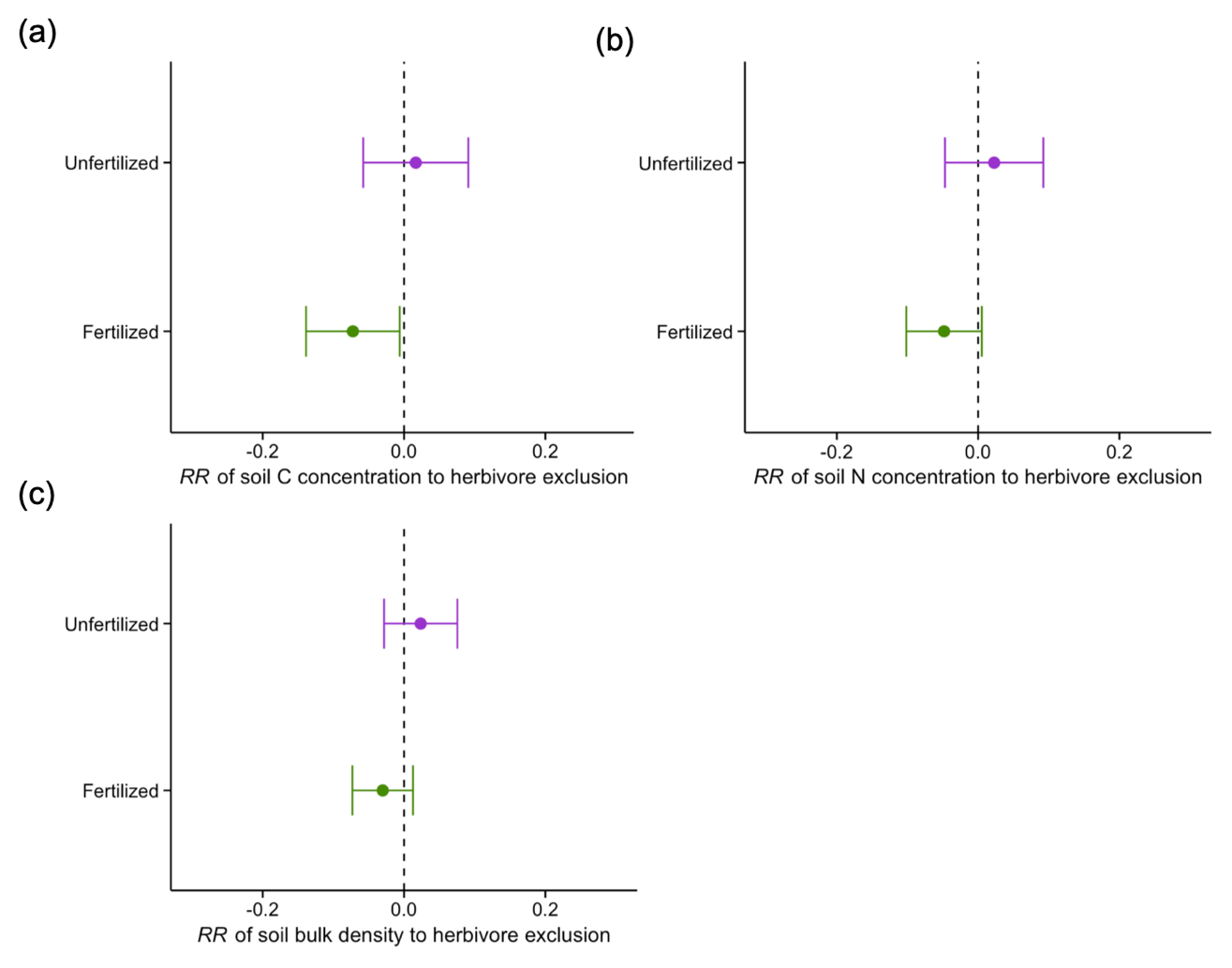
**

**Figure S1.** Log response ratios of soil C concentration (a), N concentration (b) and bulk density (c) to herbivore exclusion calculated as *RR* = ln(fenced/unfenced) for unfertilized (purple) and fertilized (NPKμ) plots (green). If *RR* = 0 herbivore exclusion had no effect on the variable, while *RR* < 0 herbivore exclusion decreased the variable, and *RR* > 0 herbivore exclusion increased the variable. Graphs show the mean *RR*s across all 22 sites (n = 63 per fertilization treatment), where points represent the mean *RR* and error bars represent the range of 95% confidence intervals. The vertical dashed line was drawn at *RR* = 0 and responses are considered significant if error bars do not overlap with zero.

**
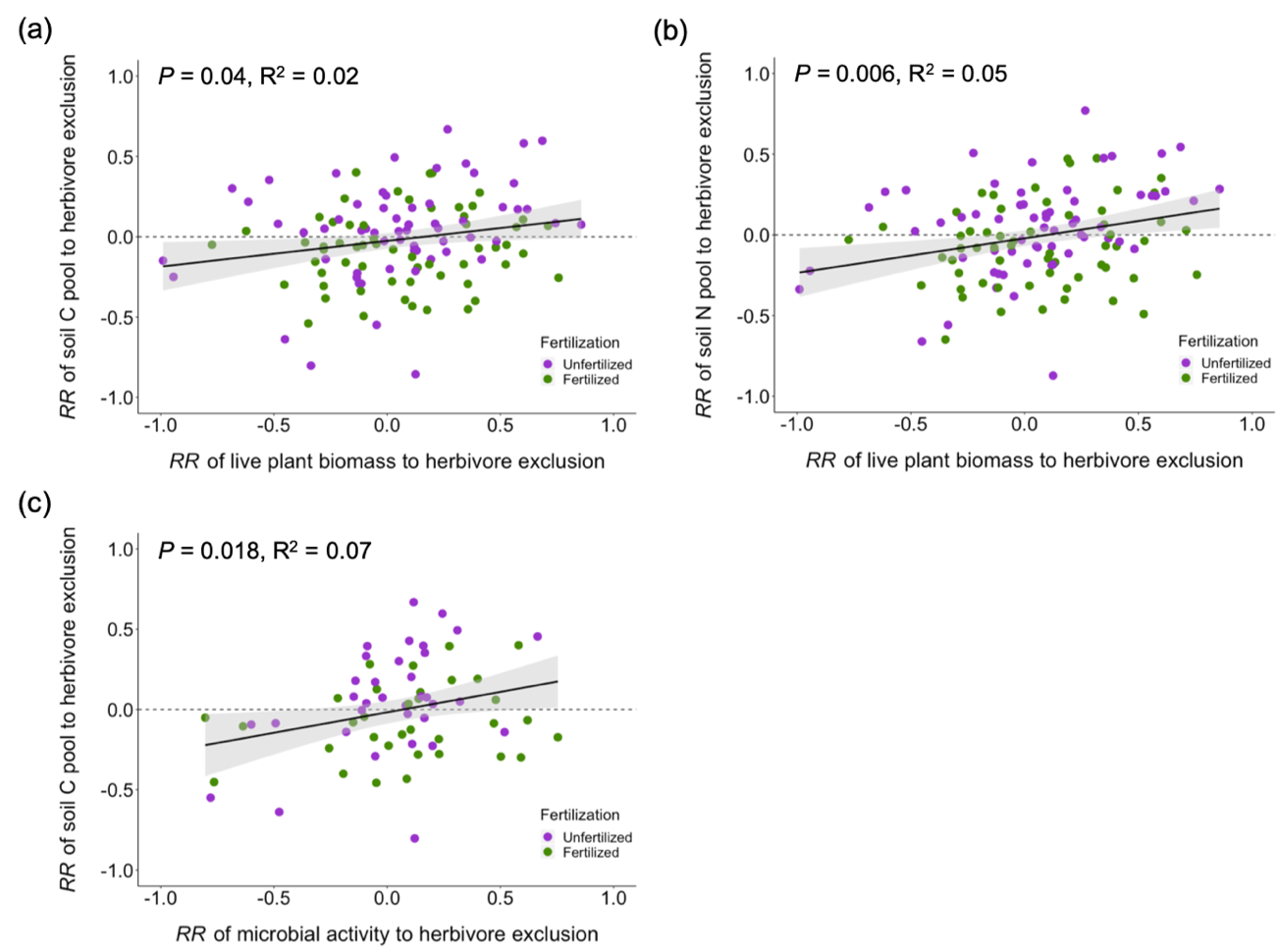
**

**Figure S2.** Relationships between the *RR* of aboveground live plant biomass and the *RR* of soil C and N pools to herbivore exclusion (a, b), and the relationship between the *RR* of microbial acitivity and the *RR* of soil C pool to herbivore exclusion (c). Purple points represent unfertilized plots, while green points represent fertilized (NPKμ) plots. All predictors showed no significant interactions with fertilization and therefore significant linear regression lines were drawn across all plots. The grey region indicates the 95% confidence interval around the regression.

**
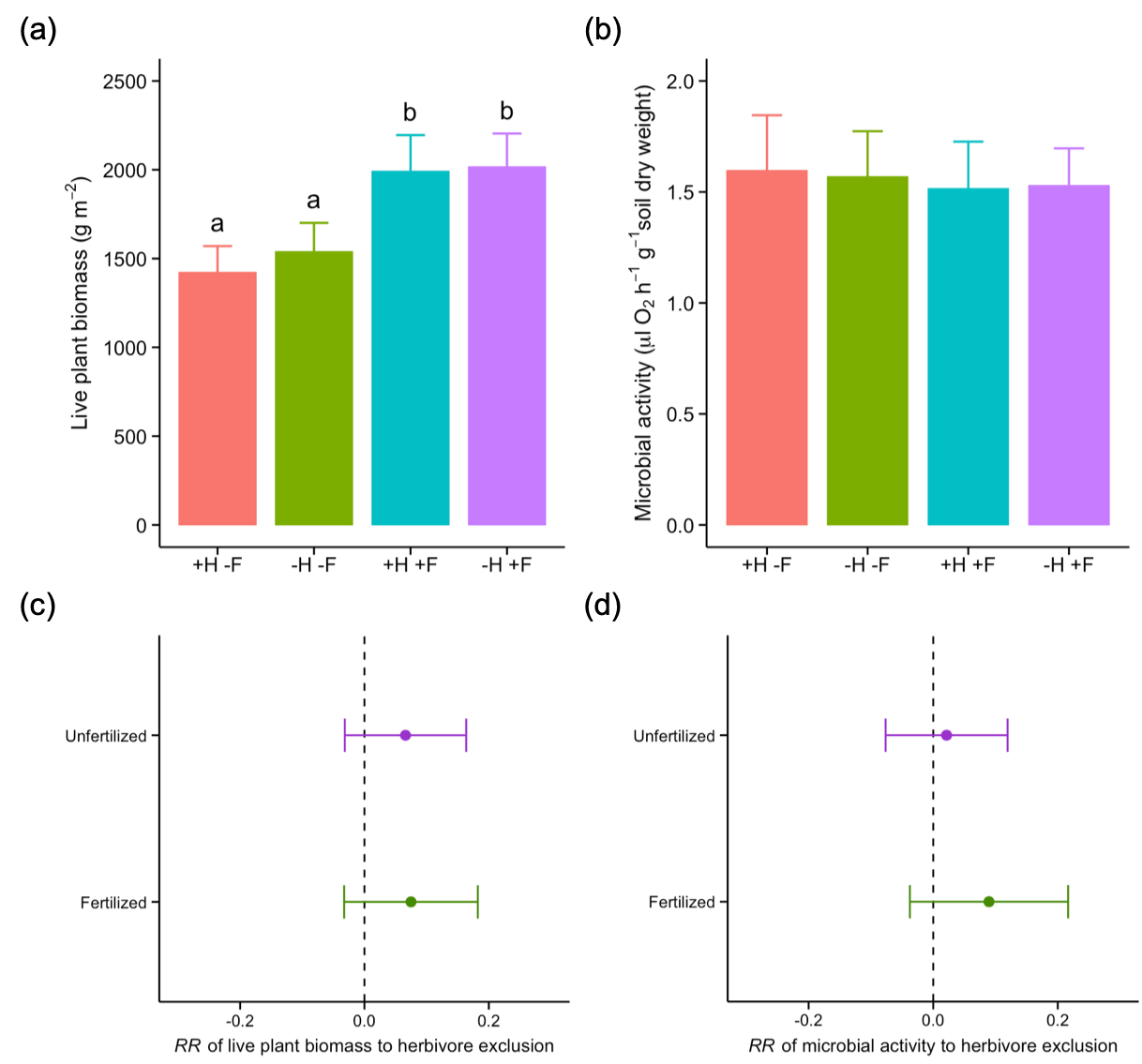
**

**Figure S3.** Effect of herbivore exclusion (+H: herbivores present, -H: herbivores excluded) and fertilization (+F: fertilized with NPKµ, -F: unfertilized) on aboveground live plant biomass (a) and microbial activity (b). Shown are sample means ± SE. Different letters indicate significant differences among the treatment means based on linear mixed models, with block nested in site as random effect and treatments as fixed. Log response ratios of aboveground live biomass (c) and microbial activity (d) to herbivore exclusion calculated as *RR* = ln(fenced/unfenced) for unfertilized (purple) and fertilized (NPKμ) plots (green). Graphs show the mean *RR*s across all 22 sites (n = 63 per fertilization treatment), where points represent the mean *RR* and error bars represent the range of 95% confidence intervals. The vertical dashed line was drawn at *RR* = 0 and responses are considered significant if error bars do not overlap with zero.

**
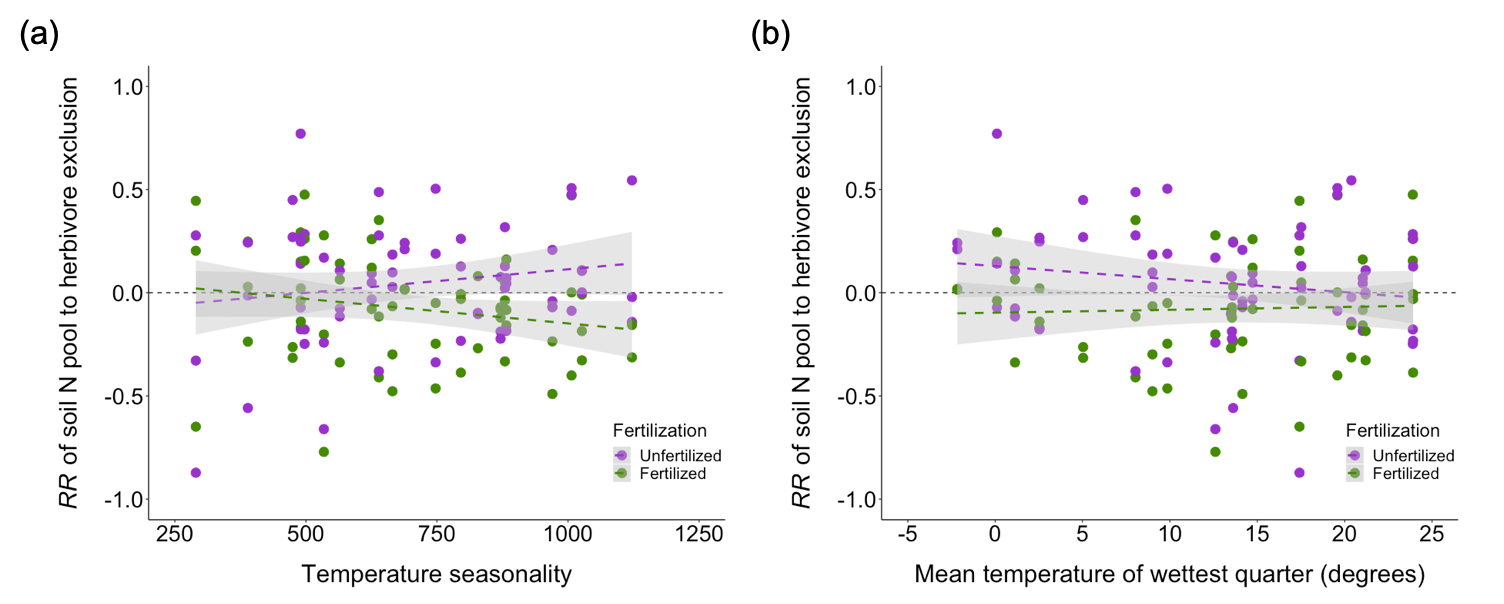
**

**Figure S4.** Relationships between temperature seasonality (a) and mean temperature of wettest quarter (b) and the *RR* of soil N pool to herbivore exclusion, which showed significant interactions with fertilization. Purple points represent unfertilized plots, while green points represent fertilized (NPKμ) plots. Non-significant linear regression lines were drawn, whereby the grey region indicates the 95% confidence interval around the regression.

**
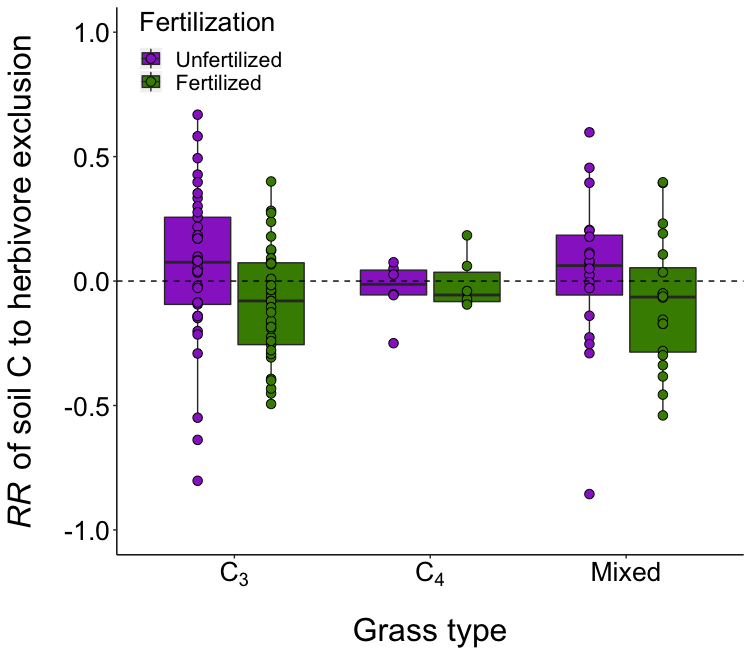
**

**Figure S5.** Log response ratios of soil C to herbivore exclusion calculated as *RR* = ln(fenced/unfenced) for unfertilized (purple) and fertilized (NPKμ) plots (green) in sites classified as C_3_-dominated, C_4_-dominated or mixed C_3_-C_4_ sites. Sites were classified based on the cover values of these grass types in each site (as in McSherry & Ritchie, 2013). This resulted in 13 sites containing only C_3_-grasses, 2 sites containing only C_4_-grasses and 7 sites that where mixed (proportion of C_4_-grasses from 0.2 to 0.9). Boxplots show the *RR*s across all blocks and include the median, while their whiskers show the minimum and maximum values. We did not find an effect of grass type on the response of soil C to herbivore exclusion (LMM, F_2,19_ = 0.01, P = 0.986).

**
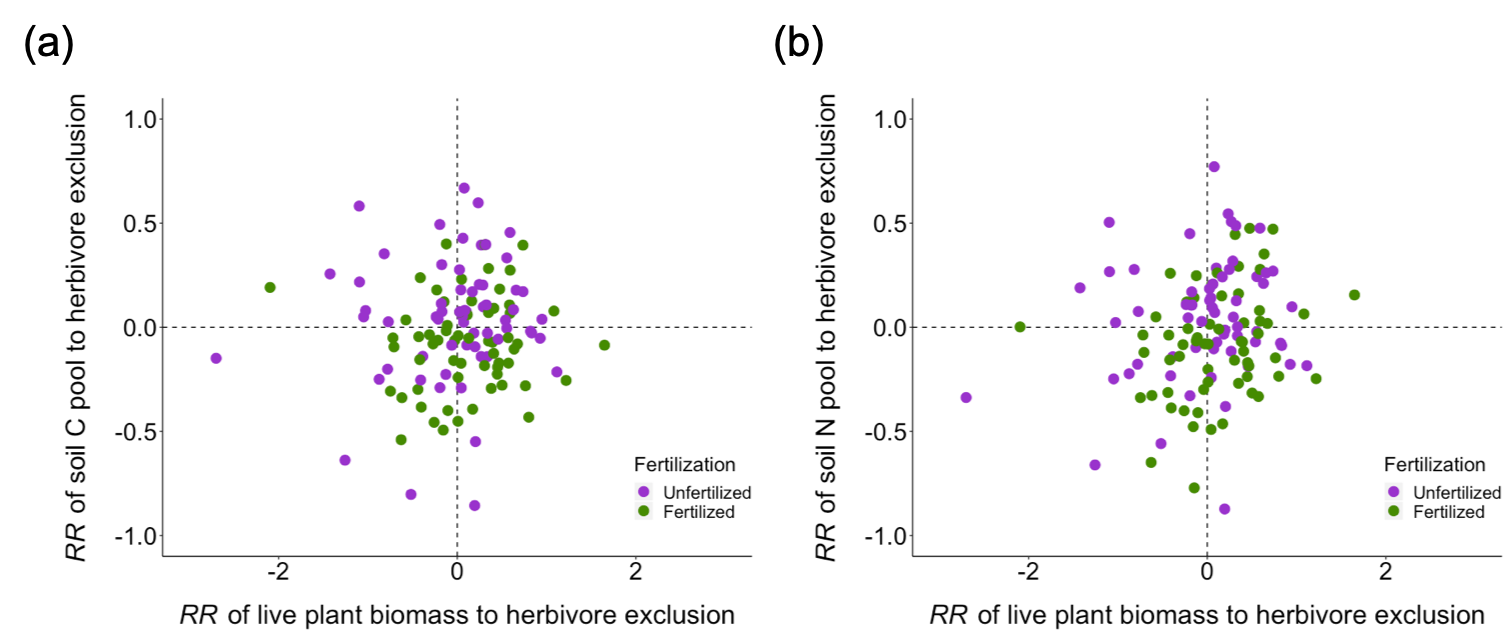
**

**Figure S6.** Scatterplots showing the *RR* of live plant biomass to herbivore exclusion as a proxy for grazing intensity and the *RR* of soil C (a) and N pools (b) to herbivore exclusion. *RR* of live plant biomass was calculated as *RR* = ln(fenced/unfenced) for unfertilized plots in each site. Live biomass data was taken after 1 year of fencing treatment as the best direct measure of grazing intensity, independent of compositional changes and species extinctions that become increasingly important after multiple years of treatments (calculated as in Seabloom et al., 2013). Purple points represent unfertilized plots, while green points represent fertilized (NPKμ) plots for the *RR* of soil C and N to herbivore exclusion.

**
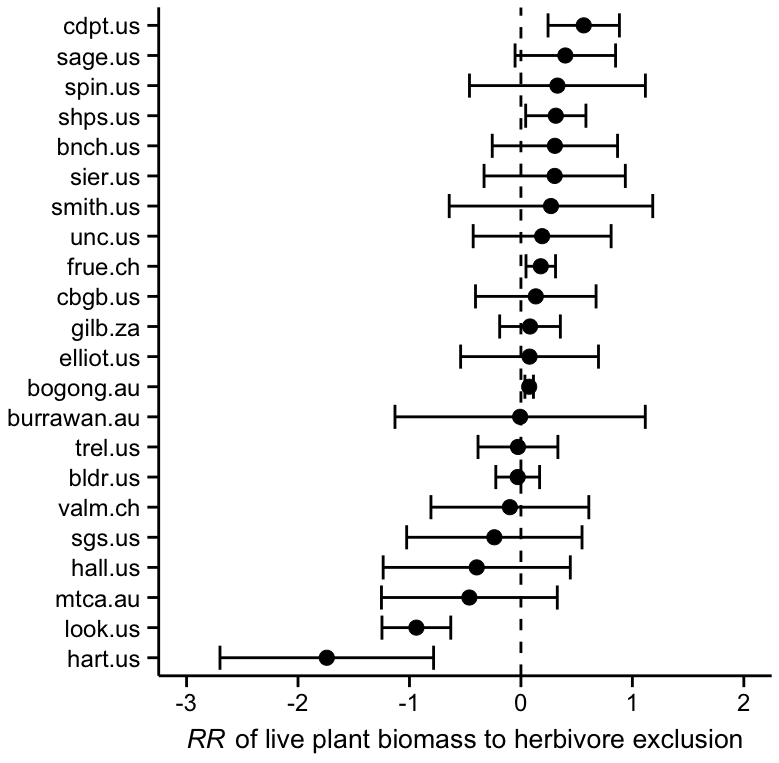
**

**Figure S7.** Log response ratios of live plant biomass to herbivore exclusion as a proxy for grazing intensity calculated as *RR* = ln(fenced/unfenced) for unfertilized plots in each site. Live biomass data was taken after 1 year of fencing treatment as the best direct measure of grazing intensity, independent of compositional changes and species extinctions that become increasingly important after multiple years of treatments (calculated as in Seabloom et al., 2013).

**
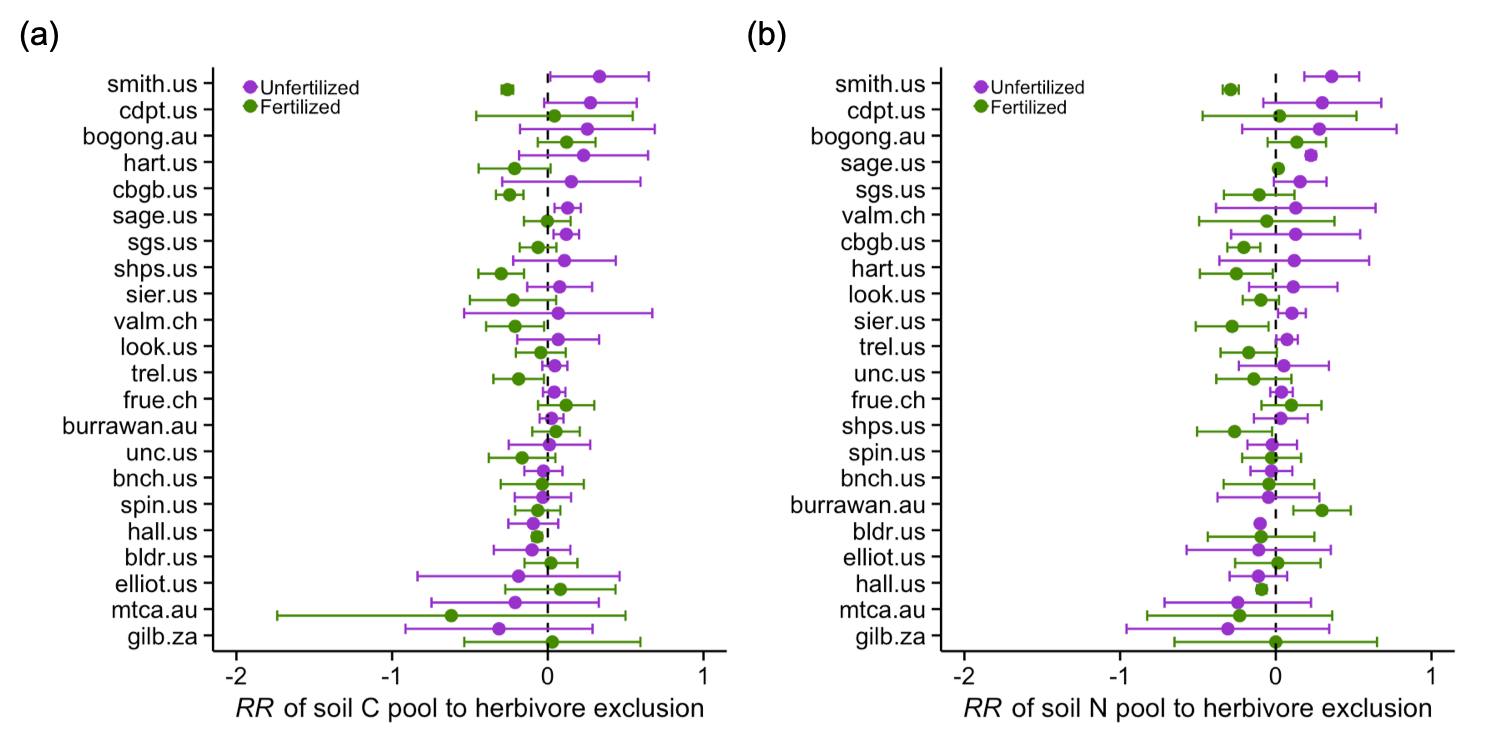
**

**Figure S8.** Log response ratios of soil C pool (a), and N pool to herbivore exclusion calculated as *RR* = ln(fenced/unfenced) for unfertilized (purple) and fertilized (NPKμ) plots (green) in each site. If *RR* = 0 herbivore exclusion had no effect on the variable, while *RR* < 0 herbivore exclusion decreased the variable, and *RR* > 0 herbivore exclusion increased the variable.

**Table S1.** Characteristics of the 22 NutNet sites included in this paper. The 12 sites with an asterisk after their number had data on soil microbial activity and biomass.

| Nr. | Continent | Habitat | Site code | Established  (year) | Measured  (year) | Latitude  (°N) | Longitude  (°E) | Elevation  (m a.s.l.) | MAP  (mm) | MAT  (°C) |
| --- | --- | --- | --- | --- | --- | --- | --- | --- | --- | --- |
| 1* | North America | Shortgrass prairie | bldr.us | 2008 | 2011 | 39.97 | -105.23 | 1633 | 487 | 9.9 |
| 2 | North America | Montane grassland | bnch.us | 2007 | 2011 | 44.28 | -121.97 | 1318 | 1618 | 6.8 |
| 3* | Australia | Alpine grassland | bogong.au | 2009 | 2012 | -36.87 | 147.25 | 1760 | 1678 | 6.0 |
| 4* | Australia | Semiarid grassland | burrawan.au | 2008 | 2012 | -27.73 | 151.14 | 425 | 643 | 18.2 |
| 5* | North America | Tallgrass prairie | cbgb.us | 2009 | 2011 | 41.79 | -93.39 | 275 | 871 | 9.3 |
| 6* | North America | Shortgrass prairie | cdpt.us | 2007 | 2011 | 41.20 | -101.63 | 965 | 456 | 9.6 |
| 7* | North America | Annual grassland | elliot.us | 2009 | 2011 | 32.88 | -117.05 | 200 | 344 | 17.7 |
| 8 | Europe | Pasture | frue.ch | 2008 | 2011 | 47.11 | 8.54 | 995 | 1546 | 7.0 |
| 9 | Africa | Montane grassland | gilb.za | 2010 | 2012 | -29.28 | 30.29 | 1748 | 943 | 14.1 |
| 10 | North America | Tallgrass prairie | hall.us | 2007 | 2011 | 36.87 | -86.70 | 194 | 1289 | 13.8 |
| 11 | North America | Shrub steppe | hart.us | 2007 | 2011 | 42.72 | -119.50 | 1508 | 259 | 7.7 |
| 12 | North America | Montane grassland | look.us | 2007 | 2011 | 44.21 | -122.13 | 1500 | 1877 | 6.9 |
| 13* | Australia | Savanna | mtca.au | 2008 | 2011 | -31.78 | 117.61 | 285 | 324 | 17.7 |
| 14 | North America | Montane grassland | sage.us | 2007 | 2011 | 39.43 | -120.24 | 1920 | 831 | 5.8 |
| 15* | North America | Shortgrass prairie | sgs.us | 2007 | 2011 | 40.82 | -104.77 | 1650 | 369 | 8.9 |
| 16* | North America | Shrub steppe | shps.us | 2007 | 2011 | 44.24 | -112.20 | 910 | 246 | 5.3 |
| 17 | North America | Annual grassland | sier.us | 2007 | 2011 | 39.24 | -121.28 | 197 | 936 | 16.3 |
| 18* | North America | Mesic grassland | smith.us | 2008 | 2012 | 48.21 | -122.63 | 62 | 605 | 10.2 |
| 19* | North America | Pasture | spin.us | 2007 | 2011 | 38.14 | -84.50 | 271 | 1152 | 12.5 |
| 20 | North America | Tallgrass prairie | trel.us | 2008 | 2011 | 40.08 | -88.83 | 200 | 992 | 11.1 |
| 21 | North America | Old field | unc.us | 2007 | 2011 | 36.01 | -79.02 | 141 | 1157 | 14.9 |
| 22* | Europe | Alpine grassland | valm.ch | 2008 | 2011 | 46.63 | 10.37 | 2320 | 681 | 0.1 |

**Table S2.** Mammalian herbivore species that are excluded by the fences at the 22 NutNet sites included in this study. This overview does not include herbivore species < 1 kg; e.g. voles (*Microtus spp.*), mice (*Peromyscus maniculatus*, *Reithrodontomys megalotis*), rats (*Dipodomys ordii*, *Sigmodon hispidus*), squirrels (*Ictidomys tridecemlineatus*, *Spermophilus beecheyi*), gophers (*Geomys bursarius*, *Thomomys spp.*) as these are likely to not be excluded by the fences.

| **Site code** | **Mammalian herbivore species** |
| --- | --- |
| Bldr.us | White-tailed deer (*Odocoileus virginianus*) |
| Bnch.us | Rocky Mountain elk (*Cervus canadensis nelson*), Roosevelt elk (*Cervus canadensis roosevelti)* |
| Bogong.au | European rabbit (*Oryctolagus cuniculus*), Hare (*Lepus europaeus*), Horse (*Equus ferus caballus*), Sambar deer (*Rusa unicolor*) |
| Burrawan.au | Black-striped wallaby (*Macropus dorsalis*), Common wallaroo (*Macropus robustus erubescens*), Cow (*Bos taurus*), Hare (*Lepus europaeus*), Eastern grey kangaroo (*Macropus giganteus*), Swamp wallaby (*Wallabia bicolor*) |
| Cbgb.us | Rabbit (*Lepus sp.*), White-tailed deer (*Odocoileus virginianus*) |
| Cdpt.us | Desert cottontail (*Sylvilagus audubonii*), Jackrabbit (*Lepus townsendi*), Mule deer (*Odocoileus hemionus*) |
| Elliot.us | Brush rabbit (*Sylvilagus bachmani*), Jackrabbit (*Lepus sp.*), Mule deer (*Odocoileus hemionus*) |
| Frue.ch | Hare (*Lepus europaeus*), Red deer (*Cervus elaphus*), Roe deer (*Capreolus capreolus*) |
| Gilb.za | Common duiker (*Sylvicapra grimmia*), Grey rhebok (*Pelea capreolus*), Hare (*Lepus capensis*), Mountain reedbuck (*Redunca fulvorufula*), Oribi (*Ourebia ourebi*), Porcupine (*Hystrix africaeaustralis*), Red rock hare (*Pronolagus sp.*) |
| Hall.us | Cottontail (*Sylvilagus sp.*), White-tailed deer (*Odocoileus virginianus*) |
| Hart.us | Horse (*Equus ferus caballus*), Jackrabbit (*Lepus sp.*), Mule deer (*Odocoileus hemionus*), Pronghorn (*Antilocapra americana*) |
| Look.us | Elk (*Cervus canadensis*), Jackrabbit (*Lepus sp.*), Mule deer (*Odocoileus hemionus*) |
| Mtca.au | European rabbit (*Oryctolagus cuniculus*), Sheep (*Ovis aries*; excluded since 2015), Western grey kangaroo (*Macropus fuliginosus*) |
| Sage.us | Mule deer (*Odocoileus hemionus*), Snowshoe rabbit (*Lepus americanus*) |
| Sgs.us | Black-tailed jackrabbit (*Lepus californicus*), Desert cottontail (*Sylvilagus audubonii*), Mule deer (*Odocoileus hemionus*), Pronghorn (*Antilocapra americana*), White-tailed jackrabbit (*Lepus townsendii*) |
| Shps.us | Black-tailed jackrabbit (*Lepus californicus*), Pronghorn (*Antilocapra americana*), Sheep (*Ovis aries*) |
| Sier.us | Jackrabbit (*Lepus sp.*), Mule deer (*Odocoileus hemionus*) |
| Smith.us | Eastern cottontail (*Sylvilagus floridanus*) |
| Spin.us | Cottontail (*Sylvilagus sp.*) |
| Trel.us | Eastern cottontail (*Sylvilagus floridanus*), White-tailed deer (*Odocoileus virginianus*) |
| Unc.us | White-tailed deer (*Odocoileus virginianus*) |
| Valm.ch | Alpine ibex (*Capra ibex*), Marmot (*Marmota marmota*), Red deer (*Cervus elaphus*), Wild alpine goat (*Rupicapra rupicapra*) |

**Table S3**. Pearson correlation matrix showing significant (*P* < 0.05) correlation coefficients (*r*) between candidate local controls of the impact of herbivore exclusion on soil C and N. *RR* total plant biomass was excluded from the multi-model inference because *r* > 0.70 (bold coefficient).

**Table S4**. Pearson correlation matrix showing significant (*P* < 0.05) correlation coefficients (*r*) between candidate environmental drivers of the impact of herbivore exclusion on soil C and N. ANN_TEMP_RANGE was excluded from the multi-model inference because *r* > 0.70 (bold coefficient).


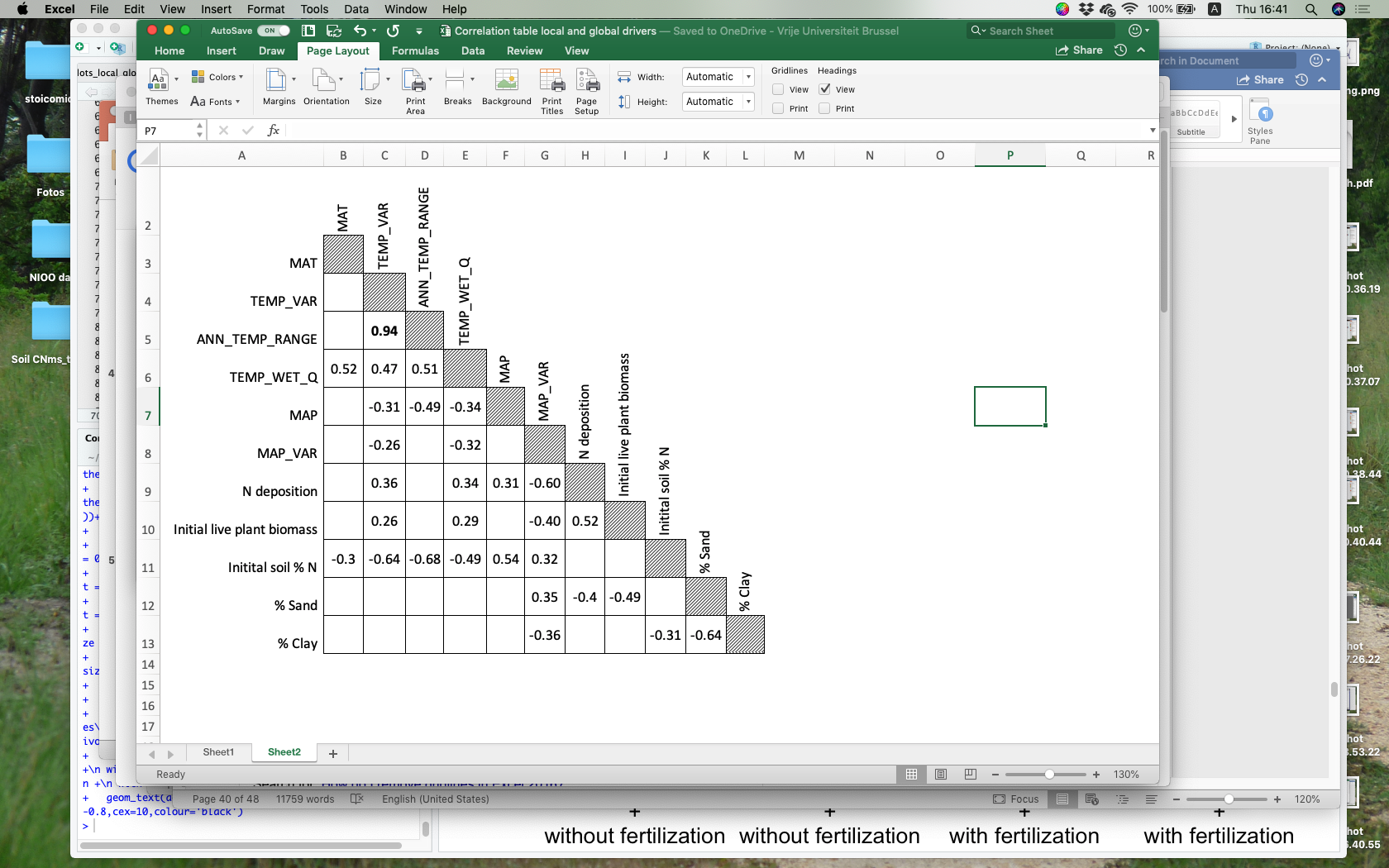


**Table S5. Effects of local controls on the response ratios of soil C and N pools and concentrations to herbivore exclusion.**

|  | *RR* of soil C pool | | | | *RR* of soil C concentration | | | |
| --- | --- | --- | --- | --- | --- | --- | --- | --- |
|  | *22 sites, n = 126* | | *+ microbial data*  *12 sites, n = 67* | | *22 sites, n = 126* | | *+ microbial data*  *12 sites, n = 67* | |
| Model-averaged R^2^ | 0.09 | | 0.22 | | 0.05 | | 0.19 | |
| Predictor variable | Estimate (SE) | *P*-value | Estimate (SE) | *P*-value | Estimate (SE) | *P*-value | Estimate (SE) | *P*-value |
| Fertilization | **-0.14 (0.05)** | **0.008** | **-0.19 (0.08)** | **0.012** | -0.09(0.05) | 0.078 | -0.09 (0.08) | 0.278 |
| *RR* live plant biomass | **0.12 (0.05)** | **0.034** | 0.09 (0.08) | 0.260 | **0.11 (0.05)** | **0.033** | 0.12 (0.08) | 0.144 |
| *RR* dead plant biomass | -0.08 (0.06) | 0.134 | -0.02 (0.08) | 0.814 | -0.04 (0.05) | 0.422 | -0.002 (0.08) | 0.980 |
| *RR* root biomass | 0.03 (0.05) | 0.609 | -0.03 (0.08) | 0.694 | 0.04 (0.05) | 0.440 | 0.05 (0.08) | 0.531 |
| *RR* microbial activity | *NA* | *NA* | **0.27 (0.09)** | **0.003** | *NA* | *NA* | **0.28 (0.09)** | **0.001** |
| *RR* microbial biomass | *NA* | *NA* | 0.08 (0.09) | 0.343 | *NA* | *NA* | 0.11 (0.09) | 0.220 |
| Fertilization:*RR* live plant biomass | -0.14 (0.11) | 0.187 | -0.01 (0.16) | 0.954 | -0.12 (0.10) | 0.232 | -0.02 (0.16) | 0.908 |
| Fertilization:*RR* dead plant biomass | 0.11 (0.11) | 0.321 | 0.25 (0.16) | 0.126 | 0.11 (0.11) | 0.306 | *NI* | *NI* |
| Fertilization:*RR* root biomass | *NI* | *NI* | *NI* | *NI* | 0.09 (0.10) | 0.383 | *NI* | *NI* |
| Fertilization:*RR* microbial activity | *NA* | *NA* | -0.18 (0.19) | 0.338 | *NA* | *NA* | -0.19 (0.17) | 0.272 |
| Fertilization:*RR* microbial biomass | *NA* | *NA* | 0.30 (0.17) | 0.089 | *NA* | *NA* | 0.22 (0.18) | 0.221 |
|  |  |  |  |  |  |  |  |  |
|  | *RR* of soil N pool | | | | *RR* of soil N concentration | | | |
| Model-averaged R^2^ | 0.12 | | 0.12 | | 0.10 | | 0.16 | |
| Predictor variable | Estimate (SE) | *P*-value | Estimate (SE) | *P*-value | Estimate (SE) | *P*-value | Estimate (SE) | *P*-value |
| Fertilization | **-0.12 (0.05)** | **0.011** | **-0.14 (0.07)** | **0.048** | -0.07 (0.04) | 0.104 | -0.04 (0.07) | 0.586 |
| *RR* live plant biomass | **0.15 (0.05)** | **0.003** | 0.14 (0.07) | 0.066 | **0.15 (0.04)** | **0.001** | **0.17 (0.07)** | **0.019** |
| *RR* dead plant biomass | -0.08 (0.05) | 0.121 | -0.02 (0.08) | 0.842 | -0.03 (0.04) | 0.446 | -0.001 (0.07) | 0.993 |
| *RR* root biomass | 0.04 (0.05) | 0.397 | 0.02 (0.08) | 0.803 | 0.05 (0.04) | 0.217 | 0.10 (0.07) | 0.183 |
| *RR* microbial activity | *NA* | *NA* | 0.14 (0.08) | 0.063 | *NA* | *NA* | **0.16 (0.08)** | **0.034** |
| *RR* microbial biomass | *NA* | *NA* | 0.05 (0.08) | 0.511 | *NA* | *NA* | 0.05 (0.08) | 0.518 |
| Fertilization:*RR* live plant biomass | -0.12 (0.10) | 0.214 | -0.06 (0.16) | 0.688 | -0.10 (0.09) | 0.237 | 0.01 (0.14) | 0.917 |
| Fertilization:*RR* dead plant biomass | 0.07 (0.10) | 0.501 | 0.26 (0.15) | 0.076 | 0.05 (0.09) | 0.551 | *NI* | *NI* |
| Fertilization:*RR* root biomass | 0.01 (0.10) | 0.950 | *NI* | *NI* | 0.06 (0.09) | 0.502 | *NI* | *NI* |
| Fertilization:*RR* microbial activity | *NA* | *NA* | -0.23 (0.15) | 0.121 | *NA* | *NA* | -0.26 (0.14) | 0.068 |
| Fertilization:*RR* microbial biomass | *NA* | *NA* | *NI* | *NI* | *NA* | *NA* | *NI* | *NI* |

*Abbreviations: RR, log response ratio to herbivore exclusion; SE, standard error; NI, variable not included in set of top models; NA, variable not added to full model. Results shown are model predictors derived after averaging submodels within 4 AIC_c_ of the best model (see Methods). All models included the random factor ‘site’. Significant predictors are in bold (P < 0.05). Effect sizes have been standardized on two SD following Gelman (2008).*

**Table S6. Effects of environmental drivers on the response ratios of soil C and N concentrations and pools to herbivore exclusion.**

|  | *RR* of soil C pool | | *RR* of soil C concentration | |
| --- | --- | --- | --- | --- |
| Model-averaged R^2^ | 0.09 | | 0.07 | |
| Predictor variable | Estimate (SE) | *P*-value | Estimate (SE) | *P*-value |
| Fertilization | **-0.14 (0.05)** | **0.007** | -0.09 (0.05) | 0.073 |
| MAT | -0.10 (0.06) | 0.078 | **-0.12 (0.05)** | **0.025** |
| TEMP_VAR | 0.01 (0.06) | 0.814 | -0.03 (0.06) | 0.591 |
| TEMP_WET_Q | -0.01 (0.07) | 0.857 | 0.03 (0.06) | 0.665 |
| MAP | 0.06 (0.06) | 0.285 | 0.06 (0.05) | 0.288 |
| MAP_VAR | 0.01 (0.06) | 0.830 | -0.01 (0.05) | 0.830 |
| N deposition | -0.03 (0.06) | 0.590 | -0.01 (0.05) | 0.905 |
| Aboveground biomass | 0.04 (0.06) | 0.540 | 0.02 (0.05) | 0.755 |
| Soil % N | -0.02 (0.07) | 0.779 | 0.03 (0.06) | 0.626 |
| Fertilization:MAT | 0.14 (0.11) | 0.197 | 0.10 (0.10) | 0.295 |
| Fertilization:TEMP_VAR | -0.21 (0.11) | 0.061 | -0.11 (0.10) | 0.276 |
| Fertilization:TEMP_WET_Q | 0.18 (0.12) | 0.130 | 0.08 (0.10) | 0.426 |
| Fertilization:MAP | 0.15 (0.11) | 0.161 | 0.03 (0.10) | 0.777 |
| Fertilization:MAP_VAR | 0.10 (0.11) | 0.343 | 0.07 (0.10) | 0.484 |
| Fertilization:N deposition | *NI* | *NI* | *NI* | *NI* |
| Fertilization:aboveground biomass | 0.10 (0.13) | 0.442 | *NI* | *NI* |
| Fertilization:soil % N | 0.16 (0.11) | 0.142 | *NI* | *NI* |
|  |  | |  |  |
|  | *RR* of soil N pool | | *RR* of soil N concentration | |
| Model-averaged R^2^ | 0.12 | | 0.10 | |
| Predictor variable | Estimate (SE) | *P*-value | Estimate (SE) | *P*-value |
| Fertilization | **-0.12 (0.05)** | **0.009** | -0.07 (0.04) | 0.099 |
| MAT | -0.09 (0.06) | 0.091 | **-0.12 (0.06)** | **0.041** |
| TEMP_VAR | -0.02 (0.06) | 0.806 | -0.07 (0.06) | 0.200 |
| TEMP_WET_Q | -0.001 (0.07) | 0.989 | 0.07 (0.07) | 0.314 |
| MAP | 0.05 (0.05) | 0.355 | 0.05 (0.05) | 0.335 |
| MAP_VAR | -0.01 (0.05) | 0.890 | -0.02 (0.05) | 0.612 |
| N deposition | -0.003 (0.06) | 0.963 | 0.01 (0.05) | 0.873 |
| Aboveground biomass | 0.03 (0.06) | 0.601 | 0.02 (0.05) | 0.745 |
| Soil % N | -0.02 (0.07) | 0.734 | 0.02 (0.06) | 0.733 |
| Fertilization:MAT | 0.17 (0.11) | 0.129 | 0.15 (0.10) | 0.118 |
| Fertilization:TEMP_VAR | **-0.28 (0.12)** | **0.018** | **-0.21 (0.10)** | **0.047** |
| Fertilization:TEMP_WET_Q | **0.26 (0.11)** | **0.016** | **0.23 (0.10)** | **0.023** |
| Fertilization:MAP | 0.10 (0.10) | 0.315 | -0.02 (0.09) | 0.858 |
| Fertilization:MAP_VAR | *NI* | *NI* | *NI* | *NI* |
| Fertilization:N deposition | *NI* | *NI* | *NI* | *NI* |
| Fertilization:aboveground biomass | 0.12 (0.11) | 0.265 | *NI* | *NI* |
| Fertilization:soil % N | 0.14 (0.10) | 0.183 | -0.11 (0.11) | 0.324 |

*Abbreviations: RR, log response ratio to grazing; SE, unconditional standard error; NI, variable not included in set of top models. Results shown are model predictors derived after averaging submodels within 4 AIC_c_ of the best model (see Methods). All models included the random factor ‘site’. Significant predictors are in bold (P < 0.05). Effect sizes have been standardized on two SD following Gelman (2008). The variables TEMP_WET_Q, MAP_VAR, N deposition, initial live plant biomass, initial dead plant biomass, initial soil % N, and initial soil pH were not included in the set of top models nor in this table.*

**Table S7**. Author contributions and site-level acknowledgments table.

| **Name** | **Contributed data** | **Developed research question** | **Analyzed data** | **Wrote paper** | **Contributed to paper writing** | **Site coordinator** | **Nutrient Network coordinator** | **Processed and paid for soil dataset** | **Site-level acknowledgments (funding, access, etc.)** |
| --- | --- | --- | --- | --- | --- | --- | --- | --- | --- |
| Judith Sitters |  | x | x | x |  |  |  |  |  |
| E.R. Jasper Wubs |  | x | x | x |  |  |  |  |  |
| Elisabeth S. Bakker |  | x |  |  | x | x |  |  |  |
| Thomas W. Crowther |  | x |  |  | x |  |  |  |  |
| Peter B. Adler | x |  |  |  | x | x |  |  | Site access: Bret Taylor of the USDA-ARS. |
| Sumanta Bagchi |  |  |  |  | x | x |  |  | Funding: MoEFCC, DBT-IISc, MHRD |
| Jonathan D. Bakker | x |  |  |  | x | x |  |  |  |
| Lori Biederman | x |  |  |  | x | x |  |  |  |
| Elizabeth T.Borer | x |  |  |  | x | x | x | x |  |
| Elsa E. Cleland | x |  |  |  | x | x |  | x |  |
| Nico Eisenhauer | x |  | x |  | x |  |  | x |  |
| Jennifer Firn |  |  |  |  | x | x |  |  |  |
| Laureano Gherardi |  |  |  |  | x | x |  |  |  |
| Nicole Hagenah |  |  |  |  | x | x |  |  |  |
| Yann Hautier |  |  |  |  | x | x |  |  |  |
| Sarah E. Hobbie |  |  |  |  | x |  | x |  |  |
| Johannes M. H. Knops | x |  |  |  | x | x |  |  |  |
| Andrew S. MacDougall |  |  |  |  | x | x |  |  |  |
| Rebecca L. McCulley | x |  |  |  | x | x |  |  |  |
| Joslin L. Moore | x |  |  |  | x | x |  |  |  |
| Brent Mortensen |  |  |  |  | x | x |  |  |  |
| Pablo L. Peri |  |  |  |  | x | x |  |  |  |
| Suzanne M. Prober | x |  |  |  | x | x |  |  | Field assistance: Georg Wiehl, site access: Denise and Malcolm French, site activity: Great Western Woodlands SuperSite of the Terrestrial Ecosystems Research Network. |
| Charlotte Riggs | x |  | x |  | x | x |  |  |  |
| Anita C. Risch | x |  |  |  | x | x |  |  |  |
| Martin Schütz | x |  |  |  | x | x |  |  |  |
| Eric W. Seabloom | x |  |  |  | x | x | x | x |  |
| Julia Siebert | x |  | x |  | x |  |  |  |  |
| Carly J. Stevens |  |  |  |  | x | x |  |  |  |
| G.F. (Ciska) Veen |  | x |  |  | x | x |  |  |  |

**Table S8**. All data contributors listed by site; site names match those in Table S1. Their effort in providing samples was critical to this study.

| **Nr.** | **Site code** | **Site PI** |
| --- | --- | --- |
| 1 | bldr.us | Kendi Davies |
| 2 | bnch.us | Elizabeth Borer, Eric Seabloom |
| 3 | bogong.au | Joslin Moore, John Morgan |
| 4 | burrawan.au | Daniel Gruner |
| 5 | cbgb.us | Lori Biederman, Stanley Harpole, Kirsten Hofmockel, Lauren Sullivan |
| 6 | cdpt.us | Johannes Knops |
| 7 | elliot.us | Elsa Cleland |
| 8 | frue.ch | Sabine Güsewell |
| 9 | gilb.za | Peter Wragg |
| 10 | hall.us | Rebecca McCulley, Jim Nelson |
| 11 | hart.us | Nicole DeCrappeo, David Pyke |
| 12 | look.us | Elizabeth Borer, Eric Seabloom |
| 13 | mtca.au | Suzanne Prober |
| 14 | sage.us | Daniel Gruner, Louie Yang |
| 15 | sgs.us | Dana Blumenthal, Cynthia Brown, Julia Klein, Alan Knapp |
| 16 | shps.us | Peter Adler |
| 17 | sier.us | Elizabeth Borer, Stanley Harpole, Eric Seabloom |
| 18 | smith.us | Jonathan Bakker |
| 19 | spin.us | Rebecca McCulley, Jim Nelson |
| 20 | trel.us | Xiaohui Feng, Andrew Leakey |
| 21 | unc.us | Amanda Koltz, Charles Mitchell, Justin Wright |
| 22 | valm.ch | Anita Risch, Martin Schütz |

**Supplement S1. Analyses and discussion of the effect of rocks on soil bulk density measurements.**

Rocks were removed from the soil cores and their weight was determined. We did not determine their volume to subtract from the volume of the soil. However, we did not have many sites that could be considered rocky; all our sites except for five (bldr.us, bnch.us, sage.us, sier.us and valm.ch) had rocks that made up <1 % of total soil weight (see the histogram below). The only site that could be considered rocky was valm.ch, where rocks made up an average of 6.3 % of total soil weight (ranging from 3.6-9.5 % in the different plots), while the other four sites had <3 % rocks. When we repeated our *RR* analyses without these rocky sites, our results remain the same; in fertilized plots the exclusion of herbivores decreased C and N pools (P = 0.02 for C and P = 0.03 for N), while herbivore exclusion had no effect without fertilization (P = 0.14 for C and P = 0.13 for N) (similar figure as Fig. 2 in the main text). We are therefore confident that our results are not strongly dependent on our soil bulk density values and we indeed found no herbivore exclusion effects on soil bulk density (Fig. S2c), indicating that the responses of soil C and N to herbivore exclusion are strongly driven by changes in soil C and N concentrations.


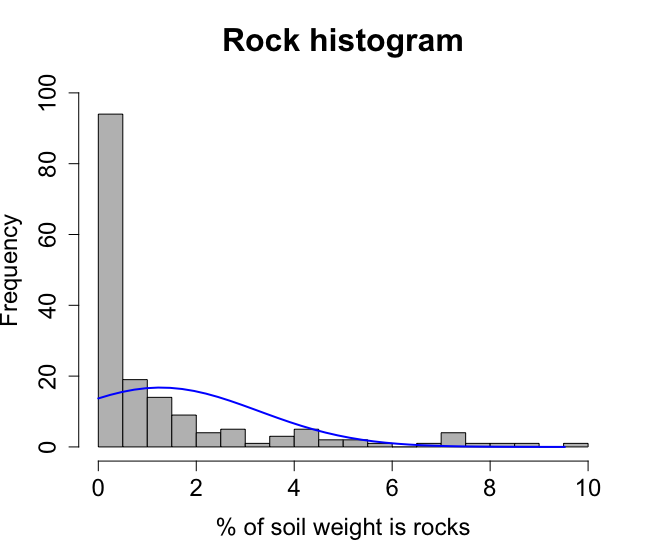

Supplement: Supplementary file 1 [file GCB-26-2060-s001.docx]
